# Ameliorative Effects of the White Jade Snail Secretion on DSS-Induced Ulcerative Colitis in Mice

**DOI:** 10.3390/cimb48101026

**Published:** 2026-10-04

**Authors:** Xiaoyan Zhang, Bin Yu, Hongyu Lu, Yi Xiong, Yixiang Li, Jianhua Yan

**Affiliations:** 1 Medical School, Guangxi University, Nanning 530004, China; zhangxiaoyangxu@outlook.com (X.Z.); yu_bin1997@163.com (B.Y.); hongyulu2000@163.com (H.L.); 2 Guangxi Center for Animal Disease Control and Prevention, Nanning 530004, China; gxxiongyi@163.com

**Keywords:** ulcerative colitis, the white jade snail, *Lissachatina fulica*, inflammatory response, intestinal barrier function, gut microbiota

## Abstract

Ulcerative colitis (UC) is a chronic relapsing inflammatory bowel disease with limited effective treatment options. The White Jade Snail Secretion (WJSS) is a biologically rich natural product with considerable therapeutic potential. UPLC–MS/MS analysis of WJSS against a standard compound spectral library and LC–MS/MS characterization of its purified protein fraction led to the identification of 88 small-molecule compounds and 782 peptide sequences, respectively. Network pharmacology analysis uncovered 240 common targets between WJSS and UC, enriched in key inflammatory pathways. A Dextran Sulfate Sodium (DSS)-induced mouse model of ulcerative colitis was employed to evaluate WJSS efficacy. WJSS treatment dose-dependently attenuated body weight loss, disease activity index elevation, and colon shortening, alongside significantly reduced histopathological scores. Mechanistically, WJSS suppressed NF-κB signaling, downregulating pro-inflammatory cytokines IL-6, TNF-α, and IL-1β. Furthermore, WJSS restored the expression of tight junction proteins (Occludin, ZO-1) and the mucin MUC2, thereby preserving intestinal barrier integrity. 16S rRNA sequencing revealed that WJSS reshaped the gut microbiota by enriching beneficial taxa (Unclassified-f-*Lachnospiraceae*) and reducing pathogenic taxa (*Campylobacter*, *Helicobacter*). Overall, WJSS alleviates DSS-induced UC through anti-inflammatory, barrier-protective, and microbiota-modulatory action, mediated by its peptides and small-molecule constituents. This study provides a multi-target mechanistic foundation for WJSS as a promising natural therapeutic candidate for UC, facilitating its clinical translation and natural product development.

## 1. Introduction

Ulcerative colitis (UC) is a chronic inflammatory bowel disease characterized by diffuse, continuous mucosal inflammation that typically originates in the rectum and extends proximally along the colon [1,2,3,4]. Clinical manifestations include persistent diarrhea, abdominal pain, rectal bleeding, and systemic complications such as fatigue and extraintestinal symptomatologies, with a relapsing-remitting disease course significantly impairing quality of life [5,6,7]. The pathogenesis of UC involves a complex interplay among genetic predisposition, environmental triggers, and aberrant immune responses to the gut microbiota, in which disruption of the intestinal epithelial barrier and microbial dysbiosis serve as central mechanistic drivers [8,9,10,11]. Current clinical treatments mainly rely on anti-inflammatory and immunomodulatory agents (e.g., corticosteroids, aminosalicylates, immunomodulators) [12,13]. However, these drugs are associated with unavoidable adverse effects, including hypersensitivity reactions and the development of glucocorticoid resistance; furthermore, a substantial proportion of patients exhibit inadequate therapeutic response or a progressive loss of responsiveness throughout treatment [14,15]. These limitations collectively compromise the long-term and more effective management approaches. Comprehensive therapeutic strategies incorporating bioactive natural products that restore intestinal barrier integrity and modulate the gut microbiota are promising candidates for the prevention and treatment of UC [16,17].

Gastropod mucus plays an essential role in locomotion, surface adhesion, defense against predators, protection from desiccation, and chemosensory-mediated behaviors such as foraging and mate recognition [18,19]. Owing to its rich biochemical composition and tunable physicochemical properties, gastropod mucus from *Achatina fulica*, *Lymnaea stagnalis*, *Helix aspersa*, etc., has attracted considerable interest because of its diverse biological activities and therapeutic potential [20,21].

Snails (class Gastropoda) comprise more than 60,000 extant species inhabiting terrestrial, freshwater, and marine environments [22,23]. Snail mucus is a viscoelastic secretion produced by the salivary glands, pedal glands, mantle, and epidermal cells of the foot [24,25]. It consists predominantly of water (approximately 90%), with the remaining fraction containing a wide range of bioactive constituents, including glycoproteins, proteoglycans, glycolic acid, allantoin, hyaluronic acid, vitamins, enzymes, antimicrobial peptides, and other biologically active molecules [22,25,26]. These constituents confer diverse pharmacological properties, including anti-inflammatory, antioxidant, antimicrobial, and tissue regenerative activities. Accordingly, snail mucus has shown therapeutic potential in the prevention and treatment of a range of chronic disorders, including inflammatory diseases, diabetic wounds, liver diseases, and certain cancers [21,27,28,29].

The White Jade Snail, an albino variant or improved breed of the giant African land snail (*Lissachatina fulica*), is an edible snail farmed in China and is a valuable source of bioactive natural products [25]. Edible snails have long been used as both food and traditional medicine in many countries owing to their high nutritional value and abundance of bioactive compounds with potential health benefits [29,30]. In our previous study, we demonstrated that White Jade Snail Secretion (WJSS) exerted significant gastroprotective effects against hydrochloric acid/ethanol-induced acute gastric ulceration, extending the current understanding of snail mucus beyond its well-established role in cutaneous wound healing [25]. These findings suggested that WJSS may exert broader protective effects on the gastrointestinal mucosal barrier. In the present study, we evaluated the therapeutic potential of WJSS in a murine model of ulcerative colitis to further assess its efficacy and provide preclinical evidence supporting its potential application in the treatment of gastrointestinal disorders.

## 2. Materials and Methods

### 2.1. Materials and Reagents

Dextran sulfate sodium (DSS) was purchased from Yeasen Biotechnology (Shanghai) Co., Ltd. (Shanghai, China); the molecular weight is 36,000–50,000 kDa. The primary antibodies for β-actin and NF-κB p65 were obtained from Proteintech Group (Wuhan, China). Antibodies against cyclooxygenase-2 (COX-2) were purchased from Jiangsu CK Biological Research Center Co., Ltd. (Nanjing, China). Horseradish peroxidase (HRP)-conjugated goat anti-rabbit and goat anti-mouse secondary antibodies were purchased from Proteintech Group (Wuhan, China). All other reagents were commercially available, of analytical or biochemical grade.

### 2.2. Preparation of WJSS

The preparation of WJSS was carried out according to the previously reported procedure [25]. The quality control standards for WJSS extraction are shown in Table S1. Briefly, the white jade snails were sourced from Lever Co., Ltd. (Luoyang, China). Mucus secretion was induced by gentle mechanical stimulation, harvested under aseptic conditions using sterile pipettes, and centrifuged immediately at 13,684× *g* for 15 min at 4 °C to pellet debris. The clarified supernatant WJSS was filtered, aliquoted into cryovials, and stored at −80 °C. Aliquots were thawed only once prior to use to prevent degradation associated with repeated freeze–thaw cycles.

### 2.3. Animal Experiments and Design

**Figure 1:**
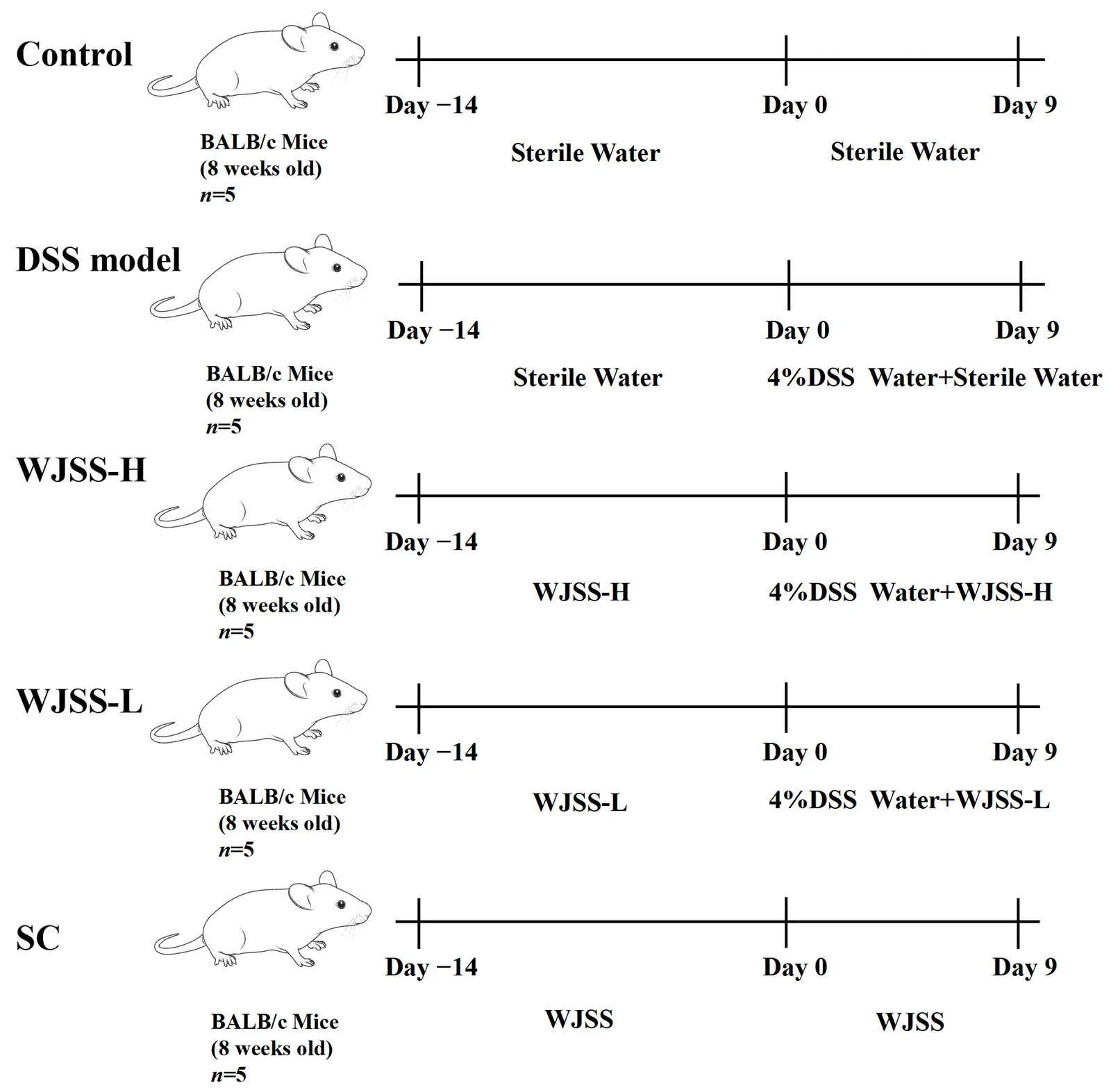
Schematic diagram of the experimental procedure.

Male BALB/c mice (6 weeks old; 20 ± 2 g) were obtained from Beijing SPF Biotechnology Co., Ltd. (Beijing, China). Animals were housed in individually ventilated cages under standard laboratory conditions and allowed to acclimatize for 7 days before the start of the experiment. The experimental design is illustrated in Figure 1. Following acclimatisation, mice were randomly assigned to five groups (*n* = 5 per group): (1) control (CON), receiving sterile water by oral gavage; (2) DSS, receiving sterile water by oral gavage; (3) SC, receiving White Jade Snail secretion (WJSS) alone; (4) WJSS-L, receiving an eight-fold dilution of WJSS followed by DSS administration. The concentrations of total polysaccharides and protein were 2.086 ± 0.106 mg/mL and 7.658 ± 0.095 mg/mL, respectively and (5) WJSS-H, receiving a four-fold dilution of WJSS followed by DSS administration. The concentrations of total polysaccharides and protein were 4.171 ± 0.213 mg/mL and 15.316 ± 0.191 mg/mL, respectively. All mice received oral gavage once daily at a dose volume of 200 μL per 20 g body weight. From Days 1 to 14, mice in the SC, WJSS-L, and WJSS-H groups received WJSS at different concentrations via oral gavage once daily, whereas mice in the CON and DSS groups received sterile water; the gavage volume was 200 μL per 20 g body weight for all groups. From Day 15 onwards, colitis was induced in the DSS, WJSS-L, and WJSS-H groups by providing 4% (*w*/*v*) DSS in the drinking water. During this period, the CON and DSS groups continued to receive sterile water by gavage, while the remaining groups continued to receive WJSS at their respective concentrations, all at a gavage volume of 200 μL per 20 g body weight. Daily WJSS or sterile water gavage was maintained throughout the DSS treatment period. Body weight and disease activity index (DAI) scores were recorded daily throughout the experiment. The DAI score primarily comprises three parameters: (1) percentage of body weight loss, (2) stool consistency, and (3) fecal bleeding. The detailed scoring criteria are in Table 1. On Day 23, all mice were euthanized by cervical dislocation, and samples were collected for further analyses. The colon was removed, laid flat without stretching or applying tension, and measured from the cecal end to the anus using a calibrated ruler; this distance was recorded as the colon length. Colon tissues and intestinal contents were collected aseptically and stored appropriately for subsequent histological, molecular, and microbiological analyses. The animal experiments were approved by the Animal Ethics Review Committee of the Scientific Research Department, Guangxi University, permit number GXU-2024-154, Approval Date: 27 December 2023.

### 2.4. Colonic Histopathology Observation

Colon tissues were fixed in 4% (*w*/*v*) paraformaldehyde at 4 °C for 48 h, followed by routine dehydration, paraffin embedding, and sectioning into 4-μm-thick sections. The sections were stained with hematoxylin and eosin (H&E) and examined using a light microscope (MI52-M, MSHOT, Guangzhou, China). Histopathological changes were evaluated using a validated scoring system based on crypt architecture, inflammatory cell infiltration, epithelial damage, and mucosal ulceration [3,30].

### 2.5. Reverse Transcription Quantitative Polymerase Chain Reaction (RT-qPCR)

Total RNA was extracted from colon tissues using the RNAprep Pure Tissue Kit (Tiangen Biochemical Technology Co., Ltd., Beijing, China) according to the manufacturer’s instructions. Total RNA was reverse-transcribed into cDNA using the FastKing RT Kit (With gDNase) (TIANGEN Biotech, Beijing, China). Briefly, 1000 ng of total RNA was used as the input per 20 μL reverse transcription reaction. Genomic DNA contamination was first removed by incubation with the kit-supplied gDNase at 42 °C for 3 min. First-strand cDNA synthesis was then carried out at 42 °C for 15 min using the kit-provided FQ-RT Primer Mix, a blend of random hexamers and oligo (dT) primers. The resulting cDNA was used as the template for quantitative reverse transcription polymerase chain reaction (RT–qPCR) to determine the mRNA expression levels of the pro-inflammatory cytokines interleukin-6 (IL-6), tumor necrosis factor-α (TNF-α), and interleukin-1β (IL-1β).

Each RT–qPCR reaction was performed in a final volume of 20 μL, containing 2 μL of cDNA template, 0.6 μL each of forward and reverse primers (10 μM), 10 μL of SYBR Green PCR Master Mix, and 6.8 μL of nuclease-free water. The amplification protocol consisted of UDG activation at 50 °C for 2 min, followed by 40 cycles of denaturation at 95 °C for 15 s, annealing at 58.5 °C for 15 s, and extension at 72 °C for 1 min. Melting curve analysis was performed following amplification to confirm the specificity of the PCR products. Relative gene expression levels were calculated using the 2^−ΔΔCt^ method, and reactions with Ct values < 35 were considered valid. Primer sequences are listed in Table 2.

### 2.6. Immunohistochemistry Staining

Paraffin-embedded colon tissue sections were deparaffinized, rehydrated through a graded ethanol series, and rinsed with phosphate-buffered saline (PBS). Antigen retrieval was performed by microwave heating in 10 mM citrate buffer (pH 6.0) for 10 min. After cooling to room temperature, the sections were blocked with 5% bovine serum albumin (BSA) in PBS for 45 min to minimize non-specific binding. The sections were then incubated overnight at 4 °C with primary antibodies against Occludin, MUC2, ZO-1, NF-κB p65, IKKβ, and COX-2 (Proteintech, China). Following three washes with PBS, the sections were incubated with horseradish peroxidase (HRP)-conjugated secondary antibodies for 30 min at room temperature. Immunoreactivity was visualized using 3,3′-diaminobenzidine (DAB) as the chromogenic substrate, followed by counterstaining with hematoxylin. The sections were subsequently dehydrated through a graded ethanol series, cleared in xylene, and mounted with neutral balsam. Images were captured using a light microscope and quantitatively analyzed in ImageJ (Version 1.53t).

### 2.7. 16S rRNA Gene Sequencing

Microbial genomic DNA was extracted from mouse colonic contents and subjected to 16S rRNA gene sequencing by Guangdong Meige Gene Technology Co., Ltd. (Shenzhen, China). The V3–V4 hypervariable regions of the bacterial 16S rRNA gene were amplified using the universal primer pair 341F and 806R. Amplicons were sequenced on the Illumina HiSeq PE250 platform (Illumina, Inc., San Diego, CA, USA) according to the standard protocols. Raw sequencing data were quality-filtered to generate high-quality effective reads. Sequences sharing ≥97% similarity were clustered into operational taxonomic units (OTUs) using UPARSE (USEARCH v10.0.240), and representative sequences from each OTU were taxonomically assigned against the SILVA database using the SINTAX classifier with a confidence threshold of 0.8. OTUs identified as contaminants were excluded prior to downstream analyses. Bioinformatic analyses were performed using R software (version 4.2.2) with the vegan and phyloseq packages. Alpha diversity was assessed using the Shannon and Simpson indices, whereas beta diversity was evaluated by principal coordinates analysis (PCoA). Differentially abundant taxa among groups were identified using linear discriminant analysis effect size (LEfSe), with a linear discriminant analysis (LDA) score > 2 and *p* < 0.05 considered statistically significant.

### 2.8. LC-MS/MS Analysis and Identified Peptides of WJSS

A 100 μL aliquot of WJSS was mixed with tris(2-carboxyethyl)phosphine (TCEP; final concentration, 10 mM), chloroacetamide (CAA; final concentration, 40 mM), and four volumes of acetonitrile. The mixture was ultrasonicated (2 s on/3 s off; total sonication time, 2 min) and then centrifuged at 12,000× *g* for 10 min. The resulting supernatant was collected and desalted using a ZipTip C18 pipette tip. The eluate was lyophilized prior to liquid chromatography–tandem mass spectrometry (LC–MS/MS) analysis. Peptide identification was performed using a Thermo Scientific Orbitrap Q Exactive HF mass spectrometer in positive ion mode. Raw mass spectrometric data were analyzed using PEAKS Studio (version 12.0) with the default search parameters against the appropriate protein database to identify and quantify peptide sequences. The physicochemical properties of the identified peptides, including hydrophobicity and net charge at pH 7.0, were predicted using the PepDraw server. The theoretical isoelectric point (pI) and molecular weight were calculated using the ExPASy Compute pI/Mw tool. The potential bioactivity of the peptides was evaluated in silico using the PeptideRanker server, whereas peptide toxicity was predicted using the ToxinPred server.

### 2.9. UPLC-MS/MS Analysis of WJSS Components and Network Pharmacological Analysis

Putative protein targets of the bioactive components in WJSS identified by UPLC–MS/MS were predicted using the SwissTargetPrediction database, and the corresponding gene names were standardized using UniProt. Ulcerative colitis (UC)-related targets were retrieved from the GeneCards and DisGeNET databases, and targets with relevance scores above the median value were selected for subsequent analysis. Shared targets between WJSS and UC were identified using Venny 2.1. A protein–protein interaction (PPI) network of the shared targets was constructed using the STRING database and visualized with Cytoscape (version 3.7.1). A component–target–disease network was subsequently established to illustrate the interactions between the bioactive constituents of WJSS, their predicted targets, and UC-associated genes. Hub targets were identified by topological network analysis in Cytoscape. Functional annotation of these hub targets was performed by Gene Ontology (GO) and Kyoto Encyclopedia of Genes and Genomes (KEGG) pathway enrichment analyses using the DAVID database.

### 2.10. Statistical Analysis

Data were analyzed using GraphPad Prism (version 9.5). Results are expressed as mean ± standard deviation (SD). Groups were analyzed using ANOVA, followed by the Least Significant Difference (LSD) post hoc test, with *p* < 0.05 considered statistically significant.

## 3. Results

### 3.1. WJSS Ameliorated DSS-Induced Colitis Symptoms in Mice

**Figure 2:**
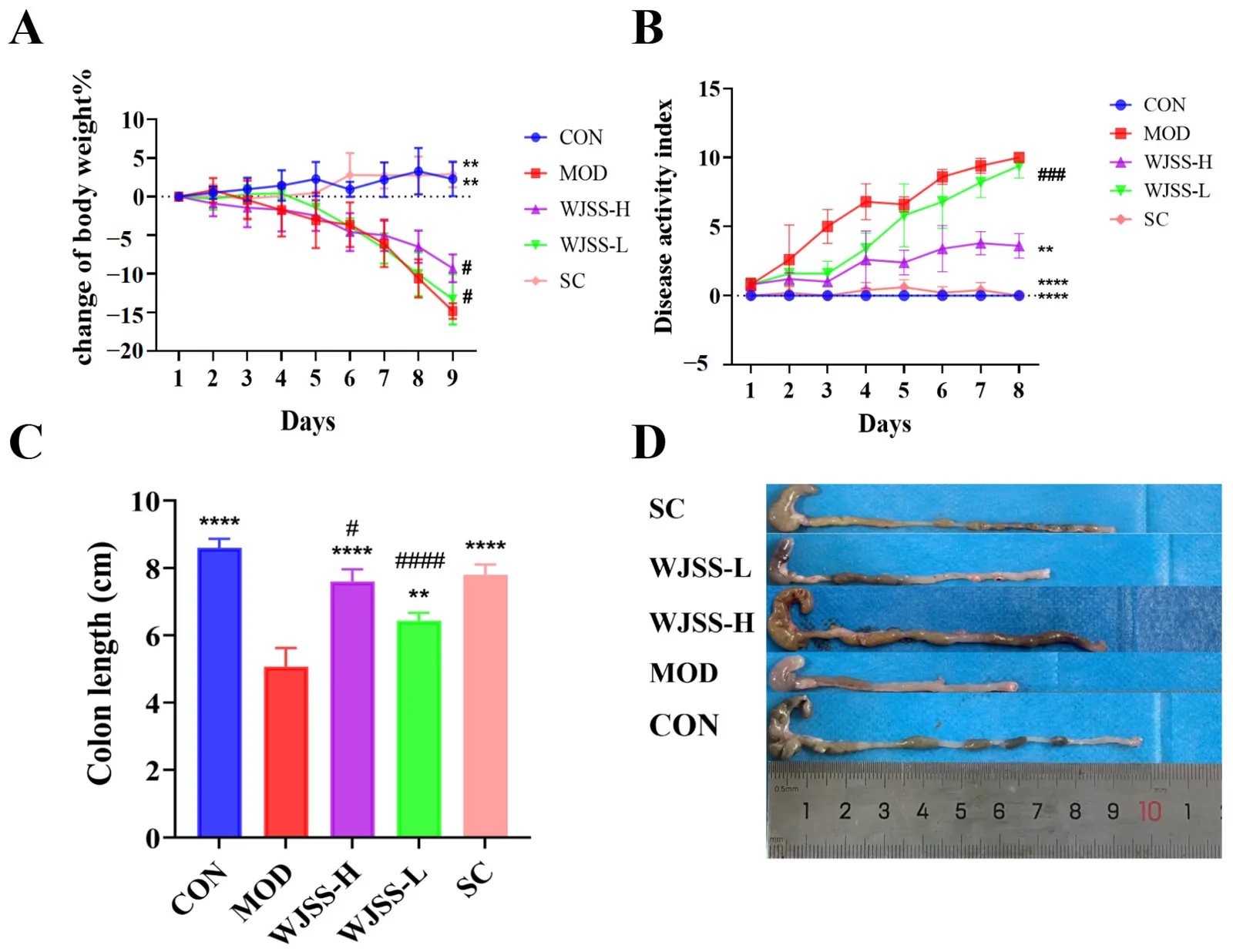
WJSS alleviates ulcerative colitis symptoms in mice. (**A**): Change in body weight. (**B**): Disease activity index of mice in each group was calculated from day 1 to day 8. (**C**): Colon length of mice in each group. (**D**): Representative images from each group of the colon. Data are expressed as mean ± standard deviation (SD). Statistics: One-way ANOVA followed by Least Significant Difference (LSD) post hoc test. * indicates comparison between each group and the MOD group. # indicates comparison between each group and the CON group. ** *p* < 0.01, **** *p* < 0.0001 vs. MOD group. # *p* < 0.05, ### *p* < 0.001, #### *p* < 0.0001 vs. CON group.

To evaluate the therapeutic efficacy of WJSS against DSS-induced UC, mice were pretreated with WJSS for 2 weeks prior to the induction of colitis by administering 4% DSS in the drinking water for 9 consecutive days. During the 9-day observation period, mice in the control (CON) and WJSS-only (SC) groups exhibited progressive body weight gain, whereas DSS-exposed groups developed hallmark UC symptoms, such as progressive weight loss, loose or watery stools, and gross rectal bleeding. Changes in body weight relative to baseline (Day 1) are presented in Figure 2A. At the end of the experiment, mice in the CON and SC groups maintained stable body weight, with the SC group showing only a negligible increase (+2.92%), confirming the absence of treatment-related adverse effects. In contrast, all DSS-exposed groups displayed significant weight loss compared with the CON group. The DSS group showed the most pronounced decline (−14.83%), whereas the WJSS intervention conferred dose-dependent protection, as the WJSS-L and WJSS-H groups lost 13.29% and 9.29%, respectively. The DAI is a validated composite score that assesses three key clinical parameters (body weight loss, stool consistency, and fecal bleeding) in murine UC models and serves as a reliable indicator of colitis severity. As shown in Figure 2B, DAI scores were significantly elevated in the DSS group compared with the CON group, confirming the successful induction of colitis. Notably, WJSS treatment markedly attenuated disease severity, as evidenced by a significant reduction in DAI scores relative to the DSS group.

**Figure 3:**
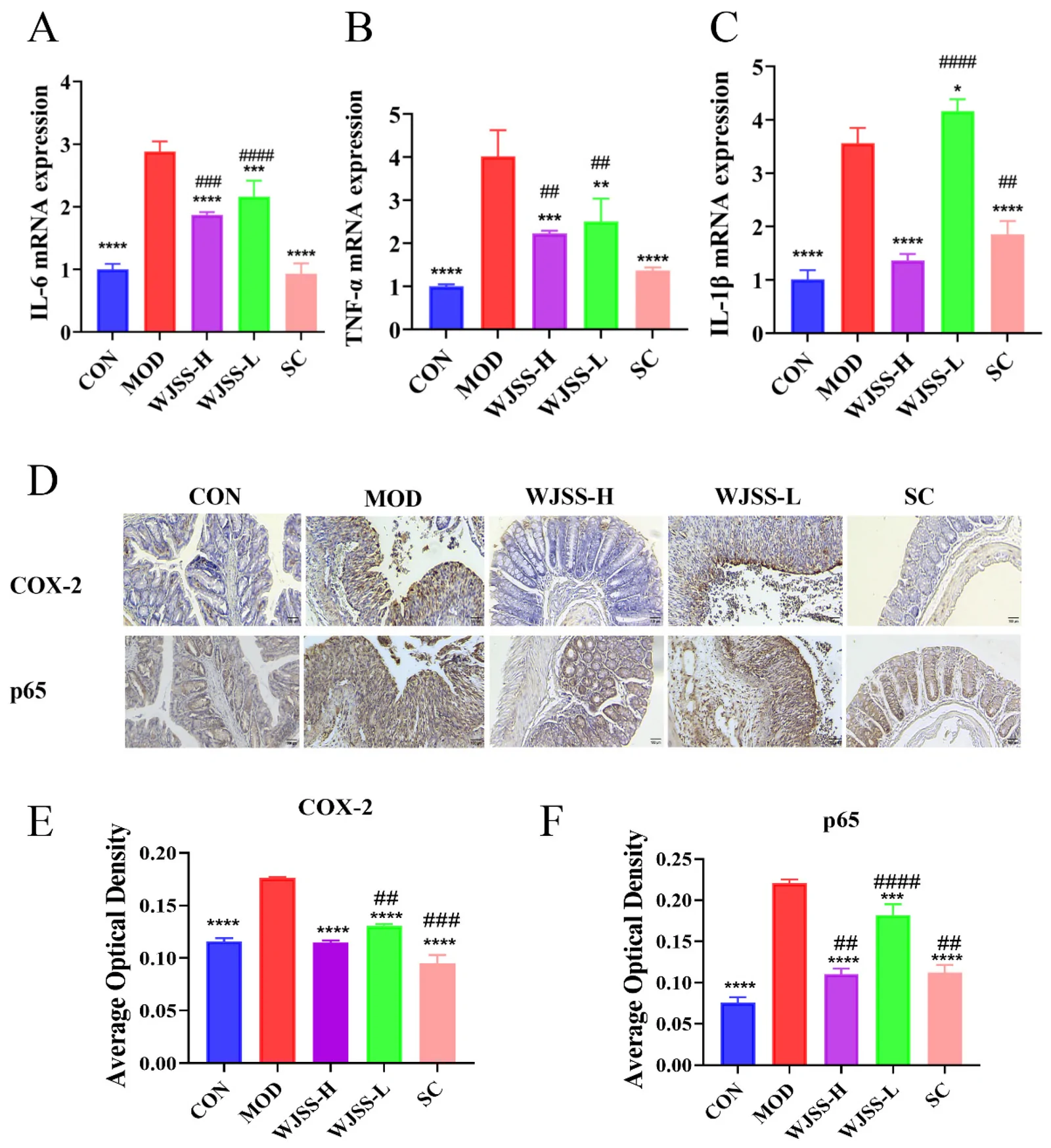
WJSS alleviates DSS-induced inflammation and inhibits the NF-κB pathway. (**A**–**C**): Levels of the pro-inflammatory cytokines IL-6 (**A**), TNF-α (**B**), and IL-1β (**C**) in colonic tissue. (**D**): Representative immunohistochemical staining images of COX-2 and NF-κB p65 in colon tissues. Scale = 100 μm. (**E**,**F**) present the quantitative analysis of the average optical density (AOD) for COX-2 and p65. Data are expressed as mean ± standard deviation (SD). Statistics: One-way ANOVA followed by Least Significant Difference (LSD) post hoc test. *n* = 3, * indicates comparison between each group and the MOD group. # indicates comparison between each group and the CON group. * *p* < 0.05, ** *p* < 0.01, *** *p* < 0.001, **** *p* < 0.0001 vs. MOD group. ## *p* < 0.01, ### *p* < 0.001, #### *p* < 0.0001 vs. CON group.

Colon length is a well-established morphological indicator of disease severity in this UC model. As shown in Figure 2C, administration of WJSS alone had no significant effect on colon length in healthy mice, whereas DSS exposure induced marked colon shortening. Strikingly, WJSS treatment dose-dependently attenuated DSS-induced colon shortening, with colon lengths of 6.43 cm and 7.60 cm in the WJSS-L and WJSS-H groups, respectively. Consistent with these macroscopic findings, H&E staining revealed severe histopathological changes in DSS-treated mice, including crypt distortion and loss, dense inflammatory cell infiltration, epithelial erosion, and submucosal edema. In contrast, WJSS treatment markedly ameliorated these pathological alterations, resulting in significantly lower histopathological scores than those in the DSS group (Figure 3A,B). Importantly, no structural abnormalities were observed in the colonic epithelium of healthy mice receiving WJSS alone, supporting its favorable safety profile under physiological conditions.

### 3.2. WJSS Attenuated DSS-Induced Colonic Inflammation

DSS administration induces colonic epithelial injury and robustly upregulates canonical pro-inflammatory cytokines. As demonstrated above, WJSS markedly ameliorates DSS-induced UC. To further elucidate its anti-inflammatory mechanism, we quantified the expression of key inflammatory mediators in colonic tissues. Consistent with previous reports on DSS-induced UC, the mRNA expression levels of IL-6, TNF-α, and IL-1β were significantly elevated in the DSS group compared with controls. Notably, WJSS treatment suppressed the transcriptional upregulation of all three cytokines (Figure 3A–C). Given the central role of the NF-κB signaling axis in driving pro-inflammatory gene expression in this experimental UC, we further examined its activation status. Immunohistochemistry revealed marked nuclear accumulation of NF-κB p65 in the DSS group, which was significantly attenuated by WJSS treatment (Figure 3D,F). Concordantly, the expression of the NF-κB-regulated downstream effector enzyme COX-2 was also substantially reduced in the WJSS-treated group relative to the DSS group (Figure 3D,E). Collectively, these findings demonstrated that WJSS exerts therapeutic effects in experimental colitis by suppressing NF-κB activation, thereby downregulating downstream pro-inflammatory mediators and mitigating mucosal inflammation.

### 3.3. WJSS Alleviated the Intestinal Barrier Injury Induced by DSS

**Figure 4:**
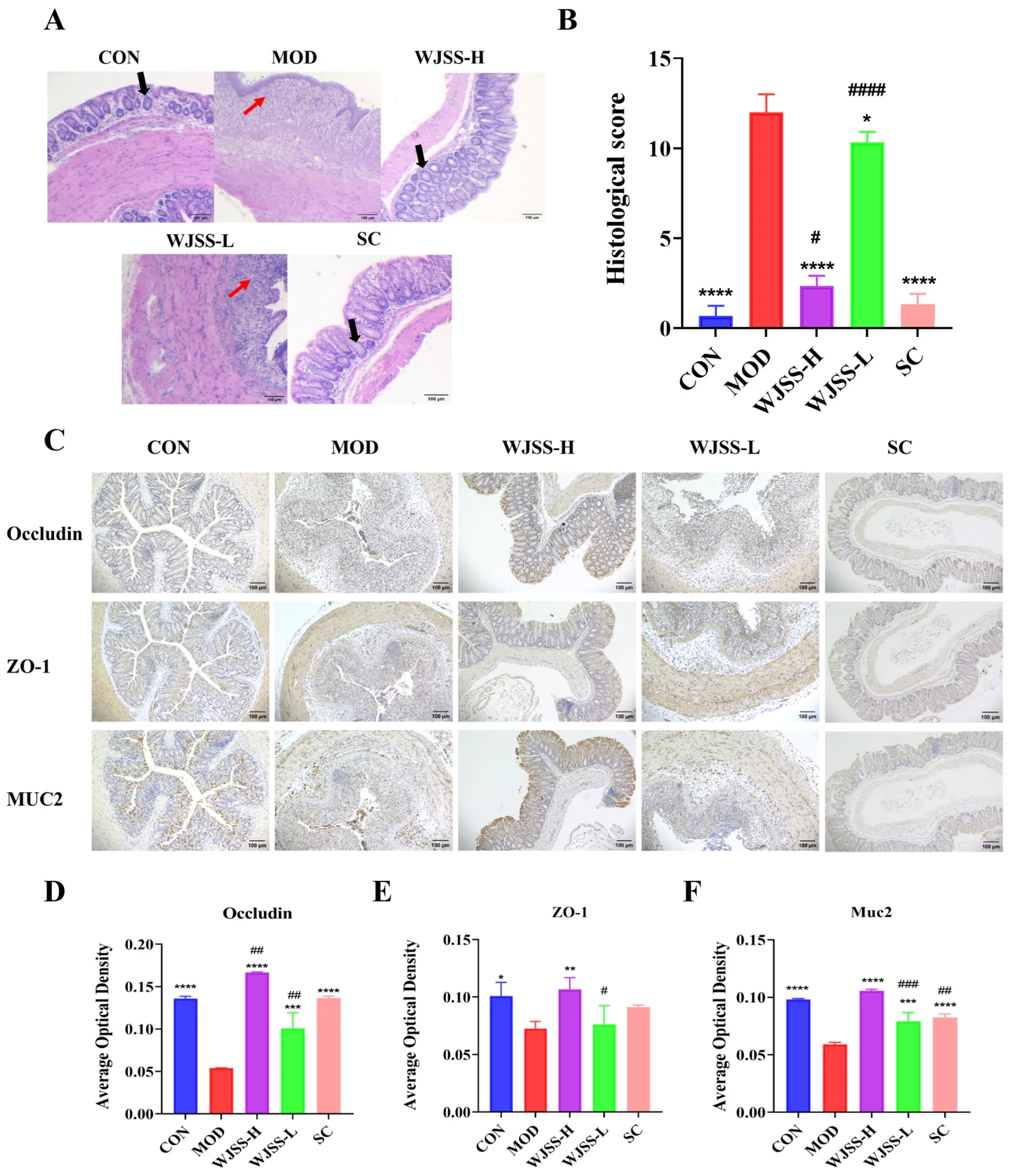
WJSS Alleviates Colon Injury and Intestinal Barrier Damage. (**A**): Hematoxylin and eosin (H&E) staining of colonic tissue. (**B**): H&E histological scoring. (**C**): Immunohistochemical analysis of Occludin, ZO-1, and MUC2 proteins in the intestine. (**D**–**F**): Analysis of the mean optical density of Occludin, ZO-1, and MUC2. Red arrows indicate sites of intestinal damage; black arrows indicate normal colonic crypt morphology with regular arrangement. Data are expressed as mean ± standard deviation (SD). Statistics: One-way ANOVA followed by Least Significant Difference (LSD) post hoc test. *n* = 3, * indicates comparison between each group and the MOD group. # indicates a comparison between each group and the CON group. * *p* < 0.05, ** *p* < 0.01, *** *p* < 0.001, **** *p* < 0.0001 vs. MOD group. # *p* < 0.05, ## *p* < 0.01, ### *p* < 0.001, #### *p* < 0.0001 vs. CON group.

To evaluate the protective effect of WJSS against DSS-induced intestinal barrier disruption, hematoxylin and eosin (H&E) staining was performed to assess colonic morphology, and immunohistochemistry was performed to assess the expression of Occludin, ZO-1, and MUC2, well-established molecular markers of epithelial barrier integrity. Representative H&E-stained images (Figure 4A) revealed intact colonic mucosa with regularly arranged crypts and minimal inflammatory cell infiltration in the CON and SC groups. In contrast, the MOD group exhibited severe architectural damage, characterized by crypt destruction and massive leukocyte infiltration. Although the WJSS-L group showed partial tissue damage, with evident inflammatory infiltrates and crypt loss, the WJSS-H group maintained relatively normal colonic architecture with well-preserved crypt alignment. As shown in Figure 4C–F, DSS administration significantly reduced the expression levels of Occludin, ZO-1, and MUC2 relative to the CON group, confirming profound barrier impairment. Intriguingly, WJSS treatment dose-dependently restored the expression of all three proteins, indicating that WJSS preserved intestinal barrier structure and function by protecting the integrity of tight junctions and the mucus layer, thereby alleviating DSS-induced damage to the colonic epithelium.

### 3.4. WJSS Intervention Reshapes the Gut Microbiota Dysbiosis in Mice with DSS-Induced Colitis

**Figure 5:**
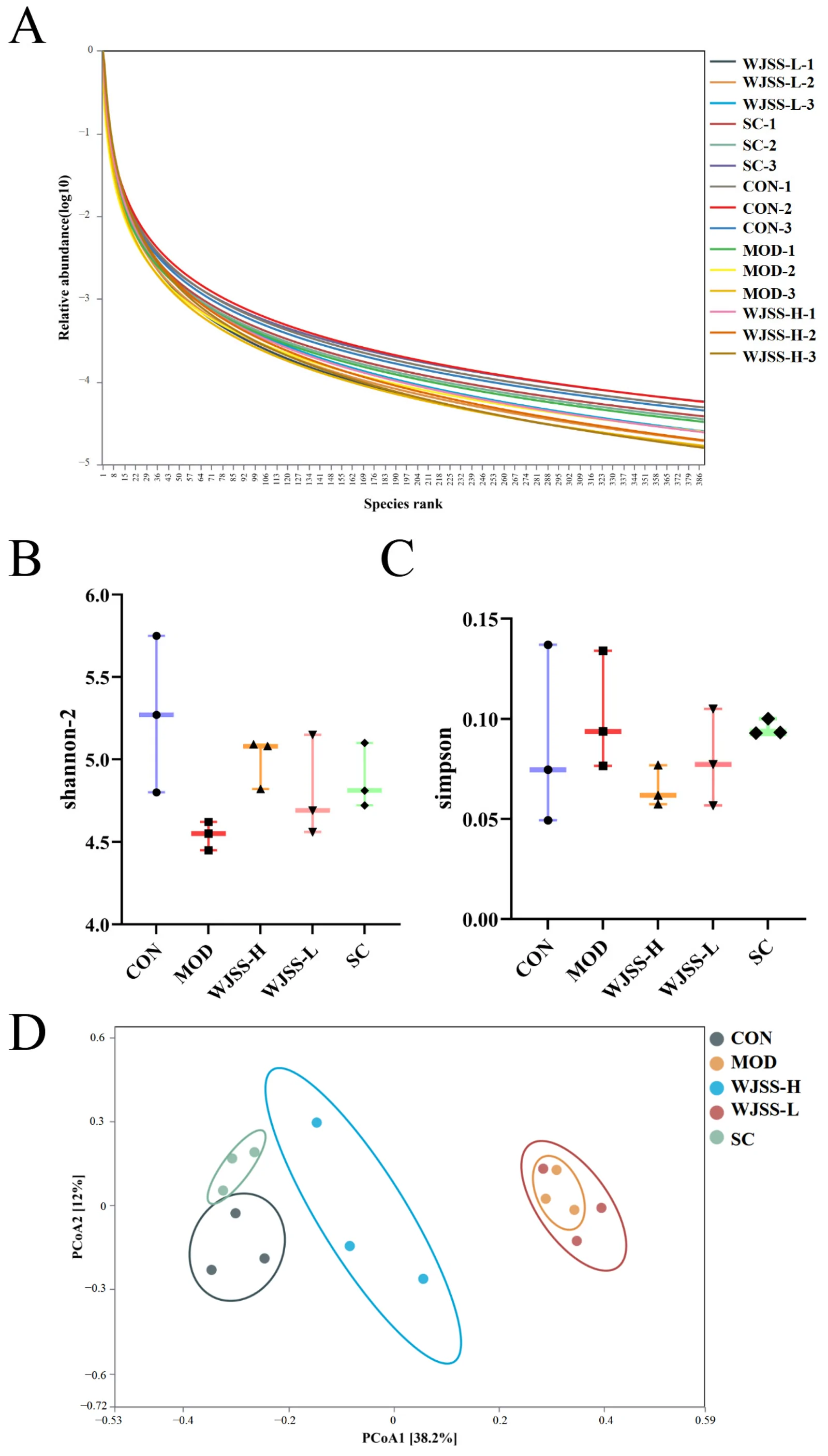
WJSS improves gut microbiota diversity and reshapes community structure. (**A**): Rank abundance curve. (**B**): Shannon-2 index. (**C**): Simpson index. (**D**): Principal Coordinate Analysis of β-Diversity.

Accumulating evidence has established a mechanistic link between gut microbiota composition and UC progression, providing a rationale for exploring microbiota-targeted therapies. In this study, intestinal luminal contents were collected from mice across all experimental groups, and the V3–V4 hypervariable region of the bacterial 16S rRNA gene was subjected to high-throughput Illumina sequencing, followed by comprehensive bioinformatic analysis. As shown in Figure 5A, the rank-abundance curve indicated differences in microbial species composition among the experimental groups. Alpha diversity was evaluated using the Shannon-2 and Simpson indices. Analysis of the Shannon-2 index revealed a statistically significant gradient in gut microbial α-diversity across the treatment groups (Figure 5B). The CON group showed the highest α-diversity, whereas the WJSS-L and WJSS-H groups exhibited intermediate levels that were comparable and showed no significant difference between them. In contrast, the MOD group showed significantly lower α-diversity than all other groups. The Simpson index reflected the dominance and evenness of the microbial communities (Figure 5C). The MOD group exhibited the lowest Simpson index, indicating pronounced dominance by one or a few bacterial taxa and, consequently, reduced community evenness; conversely, the other groups displayed comparably high and statistically similar Simpson indices, consistent with greater taxonomic evenness and community stability.

Principal coordinates analysis (PCoA) further revealed significant separation in β-diversity among the different groups (Figure 5D). PCoA1 and PCoA2 explained 38.2% and 12.0% of the total variation, respectively, accounting for a cumulative 50.2% of the variation in microbial community structure. Samples clustered by treatment group, with minimal within-group dispersion and clear between-group separation, indicating that the interventions exerted distinct, directional effects on gut microbial composition. The CON and SC groups clustered closely together, with substantial overlap in their 95% confidence ellipses, confirming that there was no statistically significant difference in β-diversity and supporting their shared status as healthy baseline microbiota. In contrast, the MOD group was shifted markedly toward PCoA1, forming a discrete cluster well separated from both the CON and SC clusters, validating the induction of obvious dysbiosis. Notably, the WJSS-H group displayed a robust leftward shift along PCoA1, occupying a position opposite the MOD group and significantly closer to the CON/SC centroid, indicating that the WJSS-H intervention effectively restored the gut microbial community toward a healthy baseline configuration.

**Figure 6:**
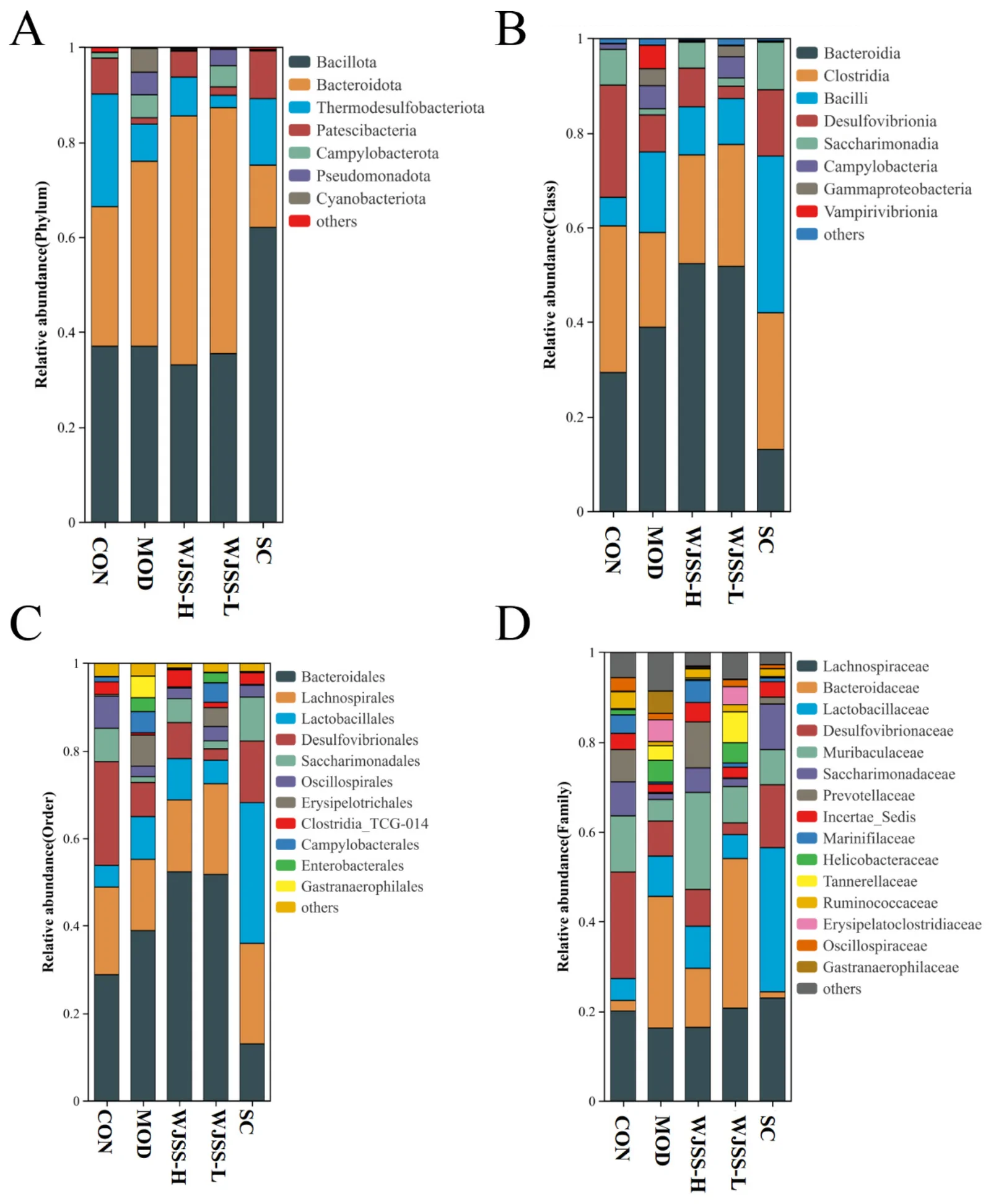
Relative abundance of microbial communities. (**A**): Relative abundance of microbial communities at the phylum level. (**B**): Relative abundance of microbial communities at the class level. (**C**): Relative abundance of microbial communities at the order level. (**D**): Relative abundance of microbial communities at the family level.

Intestinal microbiota communities differed significantly among the experimental groups across multiple taxonomic levels. At the phylum level (Figure 6A), *Bacillota* and *Bacteroidota* consistently represented the two most abundant phyla in all groups. In contrast, the MOD group displayed pronounced dysbiosis, characterized by the aberrant enrichment of *Campylobacterota*, *Pseudomonadota*, and *Cyanobacteriota*, phyla that are rarely dominant in healthy murine gut microbiomes, thereby shifting the microbial community away from physiological homeostasis. WJSS treatment reversed this dysbiotic profile, as these pathogen-associated phyla were markedly reduced or eliminated, demonstrating a dose-dependent restorative effect. Notably, the WJSS-H group not only restored overall microbial balance but also achieved the most robust reestablishment of *Bacteroidota* dominance. At the class level (Figure 6B), *Bacteroidia* remained the predominant class across all groups, underscoring its ecological stability. The abundance of *Clostridia* was significantly reduced in MOD mice but was fully restored in both WJSS-L and WJSS-H groups. Conversely, stress- and inflammation-associated classes, including *Saccharimonadia*, *Campylobacteria*, *Gammaproteobacteria*, and *Vampirivibrionia*, were enriched in MOD mice but were reduced to baseline or undetectable levels following WJSS-H treatment. The WJSS-L group exhibited partial attenuation of these changes, with residual dysbiosis features. At the order level and below (Figure 6C,D), taxonomic differences became increasingly apparent. MOD mice exhibited characteristic expansions of *Gastranaerophilales*, *Enterobacterales*, *Campylobacterales*, and *Erysipelotrichales*, taxa strongly associated with mucosal inflammation, endotoxemia, and metabolic dysfunction. WJSS treatment, particularly at the high dose, potently suppressed these detrimental orders while concomitantly enriching beneficial lineages, such as *Lachnospirales*. Similar directional changes were consistently observed at the family and genus levels, further reinforcing the coherence of WJSS-induced remodeling. Collectively, WJSS selectively enhanced commensal and beneficial taxa, including *Bacillota* and its *Clostridia*/*Lachnospirales*-associated members, while constraining opportunistic and potentially pathobiont lineages such as *Desulfovibrionales* and *Enterobacterales*. This systematic restoration of the gut microbiota across multiple taxonomic levels provides a mechanistic basis for WJSS’s beneficial effects on the homeostasis of the intestinal ecosystem.

**Figure 7:**
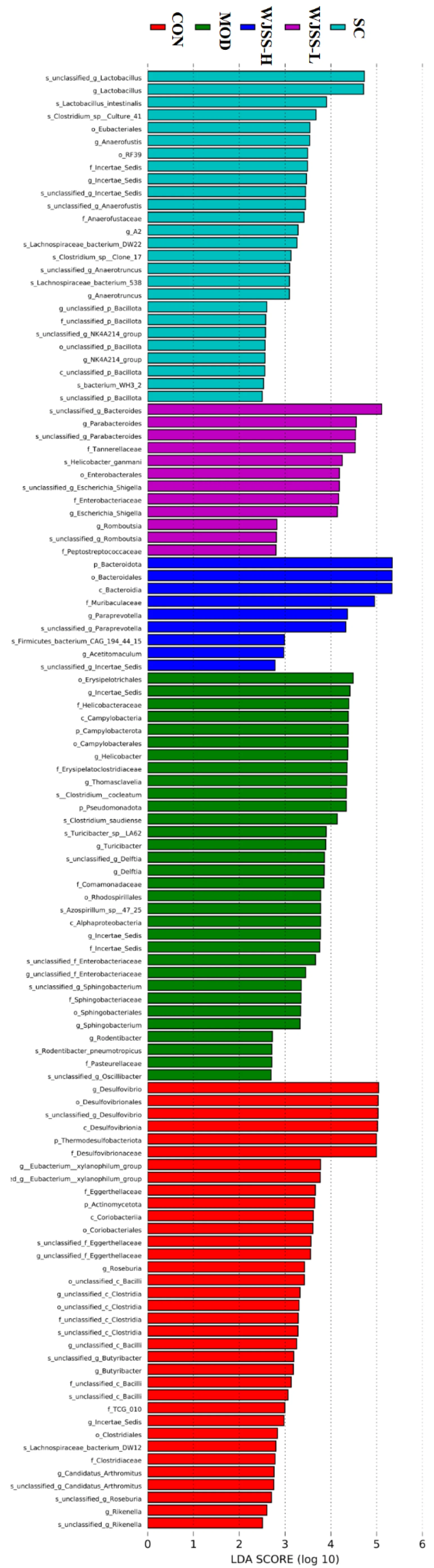
LEfSe (LDA) analysis of the gut microbiome.

**Figure 8:**
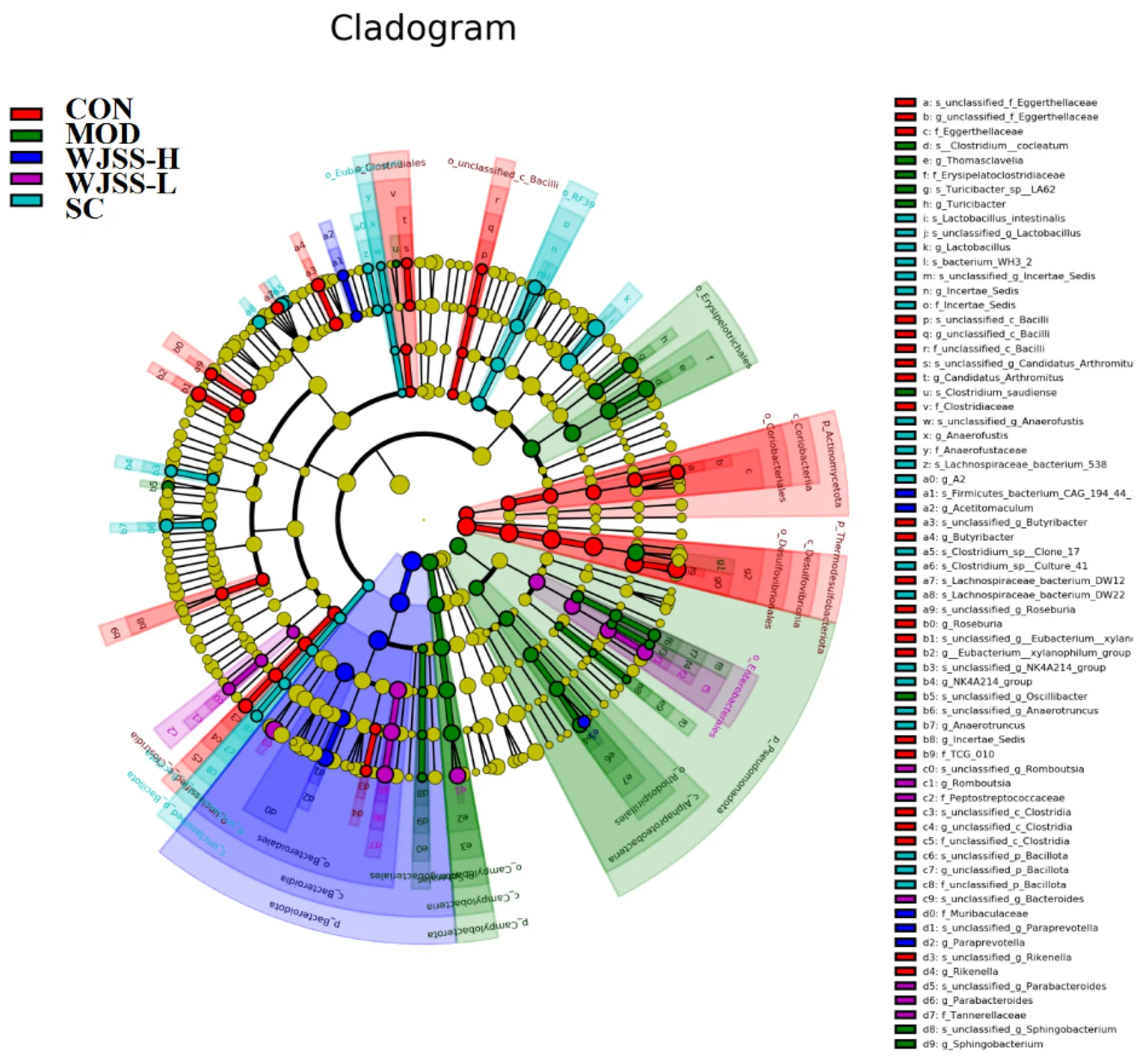
Cladogram of gut microbiota based on LEfSe analysis.

Furthermore, gut microbiota community structures across different groups were systematically compared using LEfSe analysis coupled with phylogenetic tree visualization (Figure 7 and Figure 8). This integrative approach identified statistically robust, group-specific microbial biomarkers (LDA score > 2), underscoring distinct taxonomic signatures associated with each treatment or disease state. The CON group exhibited broad, high phylogenetic representation, encompassing both beneficial commensals and several low-abundance opportunistic taxa, consistent with a resilient, homeostatic intestinal ecosystem in healthy, conventionally housed mice. In contrast, healthy mice treated with WJSS alone (SC group) showed pronounced enrichment of *Lactobacillus*, *Clostridium* clusters, *Lachnospiraceae*, and *Bacillota* (formerly Firmicutes), collectively indicating enhanced fermentative capacity, improved production of barrier-supportive metabolites (e.g., butyrate), and heightened metabolic activity within the gut ecosystem. DSS-induced colitis in the MOD group triggered marked dysbiosis, characterized by significant expansion of pro-inflammatory and pathobiont-associated lineages, including *Campylobacterota*, *Helicobacteraceae*, *Erysipelotrichales*, and *Pseudomonadota*, thereby confirming successful disease induction. The WJSS-L group showed partial restoration of the microbiota. It retained several health-associated taxa, including *Bacteroidetes* (s_unclassified_g_*Bacteroides*, g_*Parabacteroides*) and Firmicutes (*g_Romboutsia*), while concurrently harboring residual dysbiosis signatures, such as Proteobacteria (s_*Helicobacter*_*ganmani*, o_*Enterobacterales,* g_*Escherichia_Shigella*). Importantly, high-dose WJSS (WJSS-H group) induced more extensive and targeted restructuring. It significantly enriched canonical symbionts, including *Bacteroidetes* and its core lineages, f_*Muribaculaceae*, g/s_Paraprevotella and g_*Acetitomaculum*, thereby restoring taxonomic balance, functional redundancy, and host–microbe homeostasis.

### 3.5. Characterization of WJSS Peptides

Numerous studies have supported the multifaceted physiological functions of food-derived bioactive peptides [30]. Although the molecular mechanisms underlying their biological activities have not been fully elucidated, accumulating evidence suggests that the peptides’ functions largely depend on their primary structure, namely the precise sequence, amino acid composition, and overall physicochemical characteristics.

In this study, LC-MS/MS was used to profile the peptide composition of WJSS, and the physicochemical properties and potential bioactivities of the identified peptides were systematically predicted using in silico tools. A total of 782 peptide sequences, ranging from 6–32 amino acids in length, were identified in WJSS (Table S2). Five peptide characteristics, including hydrophobicity, isoelectric point (pI), net charge at pH 7, PeptideRanker score, and toxicity, were predicted using online analytical platforms. As shown in Table S3, 400 peptides were predicted to possess potential biological activity, each with a PeptideRanker score greater than 0.4. These peptides include hydrophobic, hydrophilic, and amphiphilic sequences. Additionally, their predicted pI values ranged from 3.44 to 11.01, and their net charges at pH 7 ranged from −4 to +4. According to ToxinPred, all peptides were predicted to be non-toxic, except for a single 20-amino-acid peptide (EGHEIACCIHGMPVFPHWHR). These peptides, with potential multifunctional biological activities identified in WJSS, warrant further investigation.

### 3.6. Small Molecule Compounds in WJSS Using UPLC-MS/MS and Network Pharmacological Analysis

**Figure 9:**
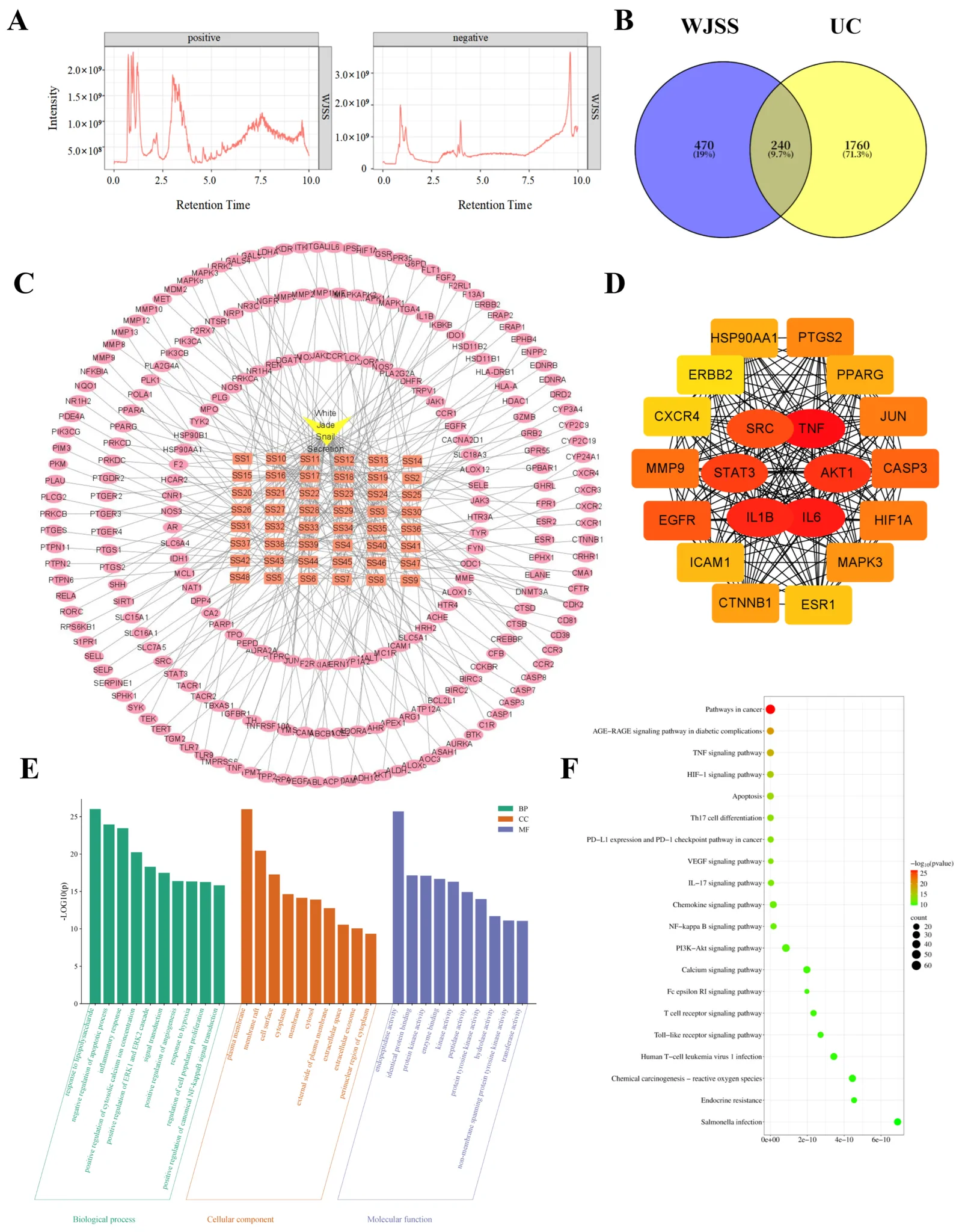
Integrated network pharmacology analysis of WJSS against ulcerative colitis. (**A**): Total Ion Chromatograms (TICs) in positive and negative ion modes. (**B**): Venn diagram showing the mapping of WJSS to targets in ulcerative colitis. (**C**): Gene network diagram showing the relationship between WJSS components, targets, and overlapping genes. Gene network diagram showing the relationship between Snail Secretion components, targets, and overlapping genes. (**D**): Interaction network of the top 20 gene targets shared between WJSS and ulcerative colitis. (**E**): Enrichment analysis of common target genes by Gene Ontology (GO) biological processes. (**F**): KEGG pathway enrichment analysis of common targets.

UPLC–MS/MS identified a total of 88 constituents in WJSS, and the corresponding positive- and negative-ion chromatograms are shown in Figure 9A and Table S4. These constituents were classified into six major superclasses: benzenoids; lipids and lipid-like molecules; organic acids and derivatives; organoheterocyclic compounds; organooxygen compounds; and phenylpropanoids and polyketides. Putative targets of the WJSS constituents were predicted using the SwissTargetPrediction database, yielding 710 candidate targets. Concurrently, a total of 2000 UC-associated targets were retrieved from the GeneCards and DisGeNET databases, with relevance scores exceeding the median by more than 4-fold. Venn diagram analysis identified 240 overlapping targets between WJSS and UC (Figure 9B).

An interaction network integrating WJSS constituents, their predicted targets, and UC-associated targets was constructed (Figure 9C). Subsequently, a protein–protein interaction (PPI) network of the shared therapeutic targets was established using the STRING database (confidence score ≥ 0.4) and further analyzed in Cytoscape. As shown in Figure 9D, hub genes were identified based on network topology. The top 20 highly interconnected nodes included TNF, IL6, IL1B, AKT1, STAT3, SRC, EGFR, CASP3, MMP9, JUN, HIF1A, PTGS2, MAPK3, CTNNB1, PPARG, HSP90AA1, ICAM1, ESR1, CXCR4, and ERBB2. These genes represent central regulators of inflammatory responses, cell survival, and tissue remodeling, processes that are critically involved in both UC pathogenesis and the predicted therapeutic actions of WJSS.

As shown in Figure 9E, Gene Ontology (GO) enrichment analysis indicated that WJSS may exert therapeutic effects against UC by modulating biological processes, cellular components, and molecular functions. The most significantly enriched biological processes included the response to lipopolysaccharide, negative regulation of apoptosis, inflammatory response, and regulation of cell population proliferation, collectively suggesting that WJSS suppresses excessive inflammation while promoting mucosal repair. Kyoto Encyclopedia of Genes and Genomes (KEGG) pathway enrichment analysis further identified several signaling pathways implicated in UC pathogenesis, including the AGE–RAGE, TNF, IL-17, NF-κB, and Toll-like receptor signaling pathways (Figure 9F). Together, these multi-level functional annotations provide convergent mechanistic evidence that WJSS alleviates colitis primarily through anti-inflammatory and barrier-protective effects. These findings are fully consistent with our previous quantitative analyses of pro-inflammatory cytokines (e.g., TNF-α, IL-6, and IL-1β) and the histopathological assessment of colonic mucosal injury and repair.

## 4. Discussion

Ulcerative colitis (UC) is a chronic, relapsing-remitting inflammatory bowel disease of unknown etiology, characterized by continuous mucosal inflammation confined to the colon and rectum [31]. Despite advances in current therapies, there remains an urgent unmet need for novel interventions that are not only safe and effective but also accessible and sustainable, making the development of such strategies a major priority in UC translational research [32,33]. In recent years, the concept of “food and medicine sharing the same origin” has demonstrated unique advantages in the prevention and treatment of chronic diseases [34,35]. Functional components derived from natural sources, owing to their multitarget activities and low toxicity, offer promising opportunities for adjunctive or alternative interventions in UC [36,37].

Snails, a widely consumed edible resource, secrete mucus rich in a variety of bioactive substances [38]. Previous studies have demonstrated that glycosaminoglycans (e.g., AFG) present in snail mucus possess potent wound-healing and antibacterial activities, highlighting their considerable potential for skin repair [28]. However, research on the role of snail mucus in intestinal inflammation remains extremely limited. Only a few studies have evaluated the protective effects of crude snail mucus extracts administered rectally or orally in rat models of UC [39,40], and the underlying mechanisms have been investigated primarily at the phenotypic level. To date, no systematic study has evaluated White Jade Snail, a strain improved through long-term artificial selective breeding, with *Lissachatina fulica* as its original parent species. Mucus from this snail species has been used as an orally administered intervention in DSS-induced UC, but its chemical composition and molecular mechanisms of action have not been comprehensively characterized.

This study presents the first systematic chemical characterization of WJSS-secreted mucus. Using LC–MS/MS, we identified 782 peptide sequences, of which 400 were predicted to have potential biological activity, with the majority being non-toxic. In addition, UPLC–MS/MS analysis identified 88 small-molecule compounds in WJSS, which were classified into six major categories: benzenoids, lipids and lipid-like molecules, organic acids and derivatives, organoheterocyclic compounds, organooxygen compounds, and phenylpropanoids and polyketides. Integrating these findings with network pharmacology analysis, we identified 240 potential target genes shared between WJSS and UC and further identified 20 core hub genes enriched in key inflammatory signaling pathways, including the TNF, IL-17, NF-κB, and Toll-like receptor signaling pathways. These findings provide the first comprehensive chemical profile of WJSS encompassing both peptide and small-molecule constituents and establish a scientific foundation for its future development as a functional food or a source of therapeutic agents.

This is the first study to profile the peptidome and metabolome of WJSS simultaneously; we benchmarked our data against published analyses of mucus from other gastropod species and against the known bioactivities of the identified molecules. At the peptide level, a substantial fraction of the identified sequences in WJSS contain the two conserved signatures of molluscan hemocyanin, the major extracellular respiratory protein of gastropods: the Pro-His-Trp-His-Arg (PHWHR) motif of the CuB domain and the Trp-Asp-Trp (WDW) motif characteristic of the CuA domain. This is consistent with previous reports that the snail mucus peptidome is dominated by fragments of the most abundant secreted and structural proteins rather than by random degradation products. The presence of bioactive proteins and peptides in the mucus of the same species complex (*Lissachatina*/*Achatina fulica*)—including the antimicrobial glycoprotein achacin and the antimicrobial peptide mytimacin-AF—further supports the view that Achatina mucus is a rich source of proteinaceous bioactives [41]. Several Gly/Pro-rich peptides additionally resemble fragments of mucin-type glycoproteins, the structural backbone of molluscan mucus, while the overall amino-acid composition (enriched in hydrophobic and aromatic residues) and the predominance of 8–20-mer peptides agree with mucus peptidomes previously reported for other gastropod species [42]. At the small-molecule level, several classes identified here are consistent with published mucus metabolomes of other land snails: free amino acids, small peptides, the osmolytes betaine and choline-type compounds, and organic acids such as citrate have all been reported in gastropod mucus [39,40]. By contrast, the tryptophan/indole metabolites (indole-3-acetic acid, indolelactic acid, 5-hydroxyindole-3-acetic acid, indole-3-carboxaldehyde and kynurenic acid), the long-chain fatty acid amides (oleamide, stearamide, palmitamide and docosanamide) and LysoPE (18:0) have not, to our knowledge, been previously reported in snail mucus and thus represent novel constituents of WJSS. Certain plant-derived terpenoids most likely originate from the snail’s vegetable diet.

Building upon the comprehensive chemical characterization of WJSS, our study further evaluated its in vivo therapeutic efficacy in a DSS-induced murine model of UC. WJSS administration conferred multi-target protective effects, ameliorating disease severity by coordinating modulation of intestinal barrier integrity, inflammatory signaling, and gut microbiota composition. First, WJSS treatment robustly restored intestinal barrier function in DSS-challenged mice. Immunohistochemical analyses confirmed significant upregulation of the tight junction proteins Occludin and ZO-1, along with marked restoration of the expression of MUC2, the principal structural glycoprotein of the protective colonic mucus layer. These findings demonstrate that WJSS preserves epithelial paracellular integrity and reinforces the physical and biochemical barrier against luminal pathogen translocation and noxious stimuli. Second, WJSS exerted potent anti-inflammatory effects in colonic tissues. RT-qPCR analysis revealed substantial suppression of the pro-inflammatory cytokine transcripts IL-6, TNF-α, and IL-1β. Mechanistically, WJSS significantly attenuated NF-κB p65 activation, thereby suppressing its activity. Consequently, the downstream effector COX-2 was downregulated, confirming functional inhibition of the NF-κB signaling axis as a central mechanism underlying the anti-inflammatory effects of WJSS.

Critically, our study employed 16S rRNA gene sequencing of the V3–V4 hypervariable region to systematically characterize WJSS-mediated modulation of the gut microbiota in DSS-induced colitis across multiple taxonomic levels, thereby providing mechanistically grounded evidence for a functional “WJSS–microbiota–host” axis. DSS administration successfully recapitulated the canonical dysbiotic phenotype associated with UC, as evidenced by a marked reduction in α-diversity, reflecting both diminished species richness and impaired community evenness, consistent with ecological destabilization dominated by opportunistic taxa [43]. The defining feature of this dysbiosis was a coordinated bidirectional shift, characterized by the expansion of inflammation-promoting and barrier-disrupting microbes accompanied by the depletion of immunomodulatory and short-chain fatty acid-producing commensals. WJSS-mediated modulation of the gut microbiota, particularly in the high-dose group (WJSS-H), extended beyond suppression of detrimental taxa and reflected a systems-level restoration of the microbial ecosystem. This was characterized by coordinated reconfiguration of the overall microbial community toward a healthy baseline, together with targeted enrichment of functionally beneficial lineages. Critically, the WJSS-H group exhibited a pronounced leftward shift along PCo1 in the PCoA analysis, with complete overlap of the 95% confidence ellipses of both the healthy CON and SC groups, demonstrating not only structural convergence but also recovery of ecological stability and functional redundancy, features strongly associated with long-term disease remission in UC. Specifically, DSS-induced depletion of *Clostridia* was robustly reversed in both the WJSS-L and WJSS-H groups. The WJSS-H group further exhibited significant enrichment of *Lachnospirales* (order) and its constituent family, Lachnospiraceae, both well-established keystone butyrate producers in the murine gut [44]. Butyrate serves as the primary metabolic fuel for colonocytes and exerts pleiotropic anti-inflammatory effects, notably by inhibiting histone deacetylases (HDACs) and suppressing NF-κB nuclear translocation [44,45]. These mechanisms plausibly underlie the WJSS-induced upregulation of the tight junction proteins Occludin and ZO-1, together with concurrent inhibition of the NF-κB signaling axis. LEfSe analysis identified f_Muribaculaceae and g_Paraprevotella as signature biomarkers that were enriched exclusively in the WJSS-H group. Both are specialized mucin utilizers. Muribaculaceae uniquely catabolize host-derived MUC2 O-glycans, and their abundance is positively correlated with mucus layer thickness and integrity [46,47,48,49]. In the present study, WJSS-driven restoration of MUC2 likely provided a critical colonization niche for these microbes; in turn, their fermentation products, such as acetate and propionate, support epithelial metabolism and reinforce mucosal barrier function, thereby establishing a self-sustaining “barrier–microbe” positive feedback loop. Moreover, the non-colitic WJSS-treated group (SC group) exhibited a modest enhancement of the baseline gut microbiota, with significant increases in *Lactobacillus* (genus), *Clostridium* clusters, and *Lachnospiraceae* (family). These findings suggest that WJSS may exert mild, selective modulatory effects on transient opportunistic bacteria through its bioactive peptides or function as a substrate with prebiotic-like properties for commensal fermenters, thereby promoting microbial resilience even under homeostatic conditions. This interpretation is consistent with the absence of histopathological abnormalities and the preservation of normal colonic architecture in SC mice.

Taken together, our study not only provides the first comprehensive characterization of WJSS components, including bioactive peptides and annotated small-molecule constituents, but also systematically demonstrates that oral administration of WJSS exerts therapeutic efficacy against DSS-induced murine UC through a coordinated tripartite mechanism involving restoration of intestinal epithelial barrier integrity, suppression of NF-κB-driven pro-inflammatory signaling, and functional reconfiguration of the gut microbiota. In contrast to previous studies, which have largely focused on dermatological applications such as cutaneous wound healing, this work pioneers the translational repurposing of WJSS for the treatment of gastrointestinal inflammation. It establishes a comprehensive, evidence-based pharmacological framework for the application of mucus derived from the food-source White Jade Snail (*Lissachatina fulica*) in UC intervention, thereby addressing an urgent need for natural product-based therapeutic strategies for inflammatory bowel disease. However, this study still has several limitations. First, the potential molecular mechanisms underlying the anti-inflammatory effects of WJSS were predicted solely through network pharmacology analysis. The screened core targets and signaling pathways have not yet been validated at the molecular level through multi-layered experiments, and further molecular biology and pharmacological studies are warranted. Second, the sample size in this animal study was relatively small, and no multi-batch comparative experiments were conducted for WJSS. Future studies should include large-scale, multi-batch investigations to comprehensively elucidate the intrinsic anti-inflammatory mechanisms of the active components in WJSS. In addition, this study has an additional limitation in this regard. In the present study, WJSS was administered to mice by oral gavage, and the intervention was not formulated as a colon-targeted delivery system. Its mechanism of action appears to involve systemic/holistic modulation of the host rather than an effect dependent on intact macromolecules reaching the colon locally; therefore, simulated gastric and intestinal fluid digestion assays were not designed as part of this study. Nevertheless, following oral gavage, the macromolecular constituents of WJSS are inevitably exposed to gastrointestinal digestive enzymes, and the observed systemic effects are more plausibly mediated by absorbed small-molecule metabolites and degradation products acting through the multi-target network described above. This constitutes a further limitation concerning the in vivo fate of these macromolecules, which could be more fully elucidated in future studies by combining in vitro digestion-stability assays with metabolomics.

## 5. Conclusions

In this study, we present the first comprehensive characterization of WJSS from the White Jade Snail (*Lissachatina fulica*) as a natural therapeutic candidate for ulcerative colitis (UC). A total of 782 peptide sequences and 88 small-molecule compounds were identified in WJSS, revealing its remarkable molecular complexity. Network pharmacology analysis demonstrated a polypharmacological profile, including the regulation of key inflammatory pathways. We further evaluated the protective effects of WJSS in a DSS-induced murine model of UC. Our findings demonstrate that WJSS suppresses NF-κB signaling, thereby reducing the expression of pro-inflammatory mediators, including IL-6, TNF-α, IL-1β, and COX-2; restores the expression of the tight junction proteins Occludin and ZO-1, as well as MUC2, thereby promoting intestinal barrier integrity; and reshapes the gut microbiota by enriching beneficial taxa while depleting pathogenic bacteria. Collectively, these results demonstrate that WJSS exerts multi-level therapeutic effects and support its potential as a safe, orally administered therapeutic candidate for UC.

## Figures and Tables

**Table 1:** Disease activity index (DAI) scoring criteria.

Score	Weight Loss (%)	Stool Consistency	Bleeding
0	0	Normal	Normal
1	1–5	Soft feces	Positive fecal occult blood
2	5–10	Loose feces	
3	10–20	Slime stool	Hematochezia
4	Greater than 20	Watery stool	

**Table 2:** Primer sequences.

Gene	5′-3′ Primer Sequences
GAPDH	F: TCAAGAAGGTGGTGAAGCAG R: GTTGAAGTCGCAGGAGACAA
IL-6	F: GGCGGATCGGATGTTGTGAT R: GGACCCCAGACAATCGGTTG
TNF-α	F: ATGAGAAGTTCCCAAATGGC R: CTCCACTTGGTGGTTTGCTA
IL-1β	F: ATGATAACCTGCTGGTGTGTGA R: TTTGTCGTTGCTTGGTTCTCC

## Data Availability

The raw data supporting the conclusions of this article will be made available by the authors upon request.

## Supplementary Materials

The following supporting information can be downloaded at: https://www.mdpi.com/article/10.3390/cimb48101026/s1.
