# Supplementary material for: Ameliorative Effects of the White Jade Snail Secretion on DSS-Induced Ulcerative Colitis in Mice
Source: Curr Issues Mol Biol. 2026 Oct 4;48(10):1026. doi: 10.3390/cimb48101026 (PMC13702899; doi:10.3390/cimb48101026)
Supplement: Supplementary file 1 [file cimb-48-01026-s001.zip › cimb-4553374-supplementary.pdf]

Title Page

Title: Ameliorative Effects of White Jade Snail Mucus on DSS-Induced Ulcerative Colitis in Mice

Xiaoyan Zhang[1†], Bin Yu[1†], Hongyu Lu[1], Yi Xiong[2], Yixiang Li[1*], Jianhua Yan[1*]

[1]Medical School of Guangxi University, Nanning 530004, Guangxi, China.

[2]Guangxi Center for Animal Disease Control and Prevention, Nanning, Guangxi, China.

*Correspondence: J. Y (Jianhuayan@gxu.edu.cn); Y. L (liyixiang_gxu@126.com)

†These authors equally shared this article.

Table S1 Essential characterization of the collected WJSS samples.

Specification	Values	Measure unit
Aspect	Clear fluid	-
Color	Whitish yellow	-
Smell	Odorless	-
PH	7.74 ± 0.14	-
Density	1.00493 ± 0.00021	$g·mL^{-1}$
Total polysaccharides	16.68545 ± 0.85189	$mg·mL^{-1}$
Protein	61.26344 ± 0.76223	$mg·mL^{-1}$
Minerals	-	-
Hg	Not detected*	$mg·kg^{-1}$
Pb	＜0.090	$mg·kg^{-1}$
Cd	Not detected*	$mg·kg^{-1}$
As	＜0.0033	$mg·kg^{-1}$

Table S2 Peptide sequences identified in WJSS.

Peptide	Length	RT
AIHSWTGGQSPYGMS	15	27.8322
ATFPQWHRLYTVQ	13	33.8621
AVFPHWHRLITVQ	13	33.5679
AVFPHWHRLLTVQ	13	33.5679
AYDPIFYLHHSNTDRIWAI	19	48.8287
AYDPIFYLHHSNTDRLWAI	19	48.8287
AYDPLFYLHHSNTDRIWAI	19	48.8287
AYDPLFYLHHSNTDRLWAI	19	48.8287
DYSAFDPFF	9	55.0956
DYSAFDPVFIIVH	13	55.0373
DYSAFDPVFIIVHS	14	55.4209
DYSAFDPVFIIVHSGMDR	18	53.6392
EFNHMTIPQLENLLK	15	48.6042
FEIAHNFIHALVGGSELY	18	50.1267
FHYEYDNLEFNGL	13	44.6546
FHYEYDNLEFNGLSIPQLAR	20	47.4087
FRLHGIGVSADVR	13	26.3068
FRLHGLGVSADVR	13	26.3068
FSMASLDYAAFDPVFFLHHSNTDR	24	50.1267
GGPLEMPWSFDRLFKY	16	49.9873
GIQPEDVFDAE	11	39.7093
HHSNVDRQFAIWQALQKFR	19	38.036
HYASYDPIFYIH	12	39.9833
HYASYDPLFYIH	12	39.9833
HYASYDPLFYLH	12	39.9833
LDYSAFDPVFIIVHS	15	55.9214
LFKYDVTNVFNKL	13	44.5474
LFNDPEFGDESFFYR	15	48.632
LFNDPEFGDESFFYRQ	16	47.8803
LFTLQIEQALHR	12	41.1293
LFTLQVEQALQR	12	43.0072
LLVVQIEQALKEK	13	42.0873
LLVVQLEQALKEK	13	42.0873
LSSLDYTLYDPIFFLH	16	56.3388
LYVEQVEEALLSH	13	49.656
LYVEQVEEALLSHGS	15	49.2699
LYVEQVEHALLEHGSSV	17	44.6002
LYVEQVEHALLEHGSSVAVPY	21	49.3811
LYVKQWEDALTAH	13	36.1838
LYVKQWEDALTAHGA	15	37.4642
LYVKQWEDALTAHGAK	16	32.4249

Peptide	Length	RT
LYVKQWEDSLTAH	13	32.7988
LYVKQWEDSLTAHGA	15	33.861
LYVVQLEQALKEKGLSI	17	49.8762
NGFQNIASFHGSPAR	15	26.125
NHFHYEYDTLEFNHM	15	32.3738
NLESLSPGEVESLRAAFL	18	51.2334
NYDRLNYQYDDLNFHGL	17	40.4182
QQRFDPNPFFSGR	13	31.6015
RVHFFIGGSGTY	12	31.2518
SLDYAAFDPVFF	12	56.685
SLDYAAFDPVFFL	13	57.5875
SLDYAAFDPVFFLH	14	55.2117
SLDYTLYDPIFFLH	14	55.5937
SLDYTLYDPIFFLHHS	16	52.0727
SLDYTLYDPIFFLHHSNVDR	20	49.0227
SLEYSAYDPIFFIH	14	52.0462
SLEYSAYDPIFFLH	14	52.0462
SLEYSAYDPLFFIH	14	52.0462
SLSSLDYTLYDPIFFLH	17	56.4708
SLSSLEYSAYDPIFFIH	17	53.6392
SLSSLEYSAYDPIFFLH	17	53.6392
SLSSLEYSAYDPLFFIH	17	53.6392
SSLDYTLYDPIFFLH	15	55.4791
STLDYSAFDPVFIIVHS	17	55.7438
STLEYTAYDPLFII	14	57.0927
STLEYTAYDPLFLL	14	57.0927
SVLGGELEMPFTFDRLY	17	54.3814
SVLGGELEMPFTFDRLYK	18	50.521
TAYDPIFYLHHSNTDRIWAI	20	49.0506
TAYDPIFYLHHSNTDRLWAI	20	49.0506
TAYDPLFYLHHSNTDRIWAI	20	49.0506
TAYDPLFYLHHSNTDRLWAI	20	49.0506
TLDYSAFDPFFIIHHS	16	53.3645
TLDYSAFDPVFIIV	14	57.5222
TLDYSAFDPVFIIVH	15	55.5074
TLDYSAFDPVFIIVHS	16	55.8594
TLEYTAYDPLFII	13	57.1623
TLEYTAYDPLFLL	13	57.1623
TNFHYEYDNLEFNGLSIPQLAR	22	47.1506
TVFDHENLGYGYDNLDIGGH	20	38.8741
VGVPYWDWTAPVRSLPSLF	19	56.7013
YDPIFYLHHSNTDRIWAI	18	48.6042
YDPIFYLHHSNTDRLWAI	18	48.6042

Peptide	Length	RT
YDPLFYLHHSNTDRIWAI	18	48.6042
YDPLFYLHHSNTDRLWAI	18	48.6042
YSLSHLEYTSYDPIFFLHHSNVDR	24	43.9027
YVEQVEEALLSH	12	38.3567
YVEQVEHALLEHGSSVAVPY	20	41.5072
YVKQWEDSLTAH	12	24.7537
EYTAYDPLFIIH	12	50.7884
EYTAYDPLFIIHHS	14	46.1966
EYTAYDPLFLIH	12	50.7884
EYTAYDPLFLIHHS	14	46.1966
EYTAYDPLFLLH	12	50.7884
EYTAYDPLFLLHHS	14	46.1966
FHYEYDSLEFNGL	13	45.1256
FRYDDDYHFK	10	21.5643
FTTHLPNVPDLNLE	14	45.8609
HHNPFHDAEIAFL	13	36.8882
HNAIHFLVGGHQTY	14	25.4327
LIVVQVEDALR	11	41.9764
LLTVQAENALRK	12	24.9335
LLVVQVEDALR	11	41.9764
LYVEQVEEALLSHGSS	16	48.9137
NDPEFGDESFFYRQ	14	38.7922
NEPLKPFSFEGFNLNKFTR	19	44.6832
QQDQGLGGFEAIAGFHGAPF	20	51.2627
SFEGFNLNKFTR	12	35.5086
SILGGETEMPFEFDRLF	17	55.428
SLEYTVYDPIFFLHH	15	52.5834
SSLEYSAYDPIFFIH	15	51.881
SSLEYSAYDPIFFLH	15	51.881
SSLEYSAYDPLFFIH	15	51.881
STLEYTAYDPLFIIHHSNVDR	21	45.6312
STLEYTAYDPLFLLHHSNVDR	21	45.6312
VGVPYWDWTAPVR	13	47.5184
YSRPIDTFDYR	11	24.7537
AIPYWDWTLPIK	12	52.6399
ALPYWDWTLPIK	12	52.6399
DYTLYDPIFFLHH	13	52.1301
FEIAHNFIHAL	11	40.0654
FHYEYDTLEFNHM	13	36.8893
GRVEFEHVDTER	12	14.4923
HSNVDRQFAIWQALQKFR	18	40.9915
LFNDPEFGSESFFYR	15	48.218
LQQDQGLGGFEAIAG	15	42.78

Peptide	Length	RT
LVVQIEQALKEK	12	32.5046
LVVQLEQALKEK	12	32.5046
LYVEQVEHALLE	12	45.571
LYVVQLEQALK	11	41.8914
QRFDPNPFFSGK	12	31.0635
SLEYSAYDPIFFIHH	15	48.1631
SLEYSAYDPIFFLHH	15	48.1631
SLEYSAYDPLFFIHH	15	48.1631
STLDYSAFDPVFIIVH	16	55.3916
DYTLYDPIFFLH	12	55.5074
FVTSDLTDPLEDLGSY	16	52.3551
ILGGPTESPWAYDR	14	36.1314
NVDRQFAIWQALQKFR	16	44.7093
RFDPNPFFSGK	11	31.3048
SFTDKIWIVWQELQK	15	52.6952
DYAAFDPVFFLH	12	54.5869
DYRNHFHYEYDTLEFNHM	18	33.8079
RYDDDYHFK	9	13.933
TAYDPIFYLH	10	45.1531
TAYDPLFYLH	10	45.1531
VFLTFPTAWWLSDAVKNPAFVVK	23	55.8617
YVEQVEEALLSHGS	14	38.1975
DYYDVWRDEVVPNPFAH	17	50.0168
NDPEFGDESFFYR	13	39.4623
SILGGETEMPFEFDR	15	47.0628
SVLGGELEMPFTFDRL	16	53.8105
EYTAYDPLFII	11	56.8003
EYTAYDPLFLI	11	56.8003
EYTAYDPLFLL	11	56.8003
LYVEQVEEALLSHGSSVAVPY	21	53.0053
FHYEYDNLEFN	11	36.8084
HYQYDDLNFH	10	25.3814
FLHNRVHFFIGGSGTY	16	33.6472
YDDDYHFH	8	16.6709
VFAGFLLKGIK	11	40.5012
HIDDANKDTVR	11	5.7982
SISSLDYSAFDPVFFLH	17	55.3916
SLDYAAFDPVFFLHHS	16	51.8668
SLSSLDYSAFDPVFFLH	17	55.3916
SAGEIESLRSAFLAIQ	16	48.6042
DYSAFDPFFIIHHS	14	52.7524
SLSYSAFDPIFYIH	14	52.7524
SLSYSAFDPIFYLH	14	52.7524

Peptide	Length	RT
TLEYTAYDPLFIIH	14	52.7236
TLEYTAYDPLFLLH	14	52.7236
SYSAFDPIFYIH	12	52.074
SYSAFDPIFYLH	12	52.074
FDPNPFFSGK	10	36.893
FTLQIEQALHR	11	35.6179
EYSAYDPIFFIHH	13	44.4925
EYSAYDPIFFLHH	13	44.4925
EYSAYDPLFFIHH	13	44.4925
HHNPFHDAEIA	11	16.2505
LYVEQVEHAL	10	35.7525
ISSLDYSAFDPVFFLH	16	55.124
LDYTLYDPIFFLH	13	55.6238
LSSLDYSAFDPVFFLH	16	55.124
HNALHSWLGGR	11	16.5667
FRLHGIGVSADVRVK	15	26.3916
FRLHGLGVSADVRVK	15	26.3916
QFAIWQALQKFR	12	48.632
AFDPVFIIVH	10	51.0095
LGLPFPNLPYL	11	57.258
LRKPLPPFSL	10	34.5285
SAFDPVFIIVHS	12	51.7958
LNYQYDDLNFHGL	13	42.8656
YSISSLDYSAFDPVFFLHHANTDR	24	50.5543
YSLSSLDYSAFDPVFFLHHANTDR	24	50.5543
SYSAFDPIFY	10	51.5377
SLDYSAFDPVFFLHHA	16	51.2615
LFNDPEFGDESFFY	14	54.1553
FLIHNIGLSADVE	13	42.4175
RFDPNPFFSGR	11	31.9221
AILGGPLEMPWSFDR	15	50.6234
YSRPADTFDYR	11	19.8382
FRYDDDYH	8	15.3466
GGPTESPWAY	10	33.7548
SLDYTLYDPIFFLHH	15	52.583
LLVVQIEQALK	11	46.2775
LLVVQLEQALK	11	46.2775
EYTSYDPIFFLH	12	50.6234
EYTSYDPLFFLH	12	50.6234
LIVVQVEDALRRR	13	34.3597
LLVVQVEDALRRR	13	34.3597
GGPLEMPWAF	10	54.2123
SVLGGELEMPFTFDR	15	50.9271

Peptide	Length	RT
LIHNIGLSADVE	12	32.5288
HNTIHFLVGGR	11	21.8768
EYTAYDPI	8	35.2163
EYTAYDPL	8	35.2163
VHFFIGGSGTY	11	37.1597
AVFPHWHR	8	18.9227
LYVEQVEEALLSHG	14	50.0151
KNVESLSPLEGY	12	32.399
SILGGETEMPFEFDRL	16	51.7667
YTSYDPIFF	9	51.5972
YTSYDPLFF	9	51.5972
GGETEMPFEFDRLFRI	16	52.6668
LRKPLPPFS	9	24.2299
DYAAYDPVFFLHHT	14	50.2497
LIVVQVEDALRR	12	37.4647
LLVVQVEDALRR	12	37.4647
HGAPARCPDPDHPTVACCQHGMPTFPHWHR	30	46.6729
FHYEYDNLE	9	27.4917
FIIHHSNVDRQFAIWQAL	18	43.9597
FLIHHSNVDRQFAIWQAL	18	43.9597
FLLHHSNVDRQFAIWQAL	18	43.9597
SYVHTPLKPF	10	28.3562
HLPNVPDLNLE	11	39.6264
FTLQVEQALQR	11	36.7819
GSSKEMPWTNEILFKY	16	44.9055
PSFPHWHR	8	18.3469
IHSWLGGHAK	10	12.624
LHSWLGGHAK	10	12.624
GRVEFEHVDTE	11	17.8244
YHFVDHIVAV	10	33.1171
DYSAFDPVFFLHHA	14	50.5132
IIFEPAHGA	9	26.2538
SILGGETEMPFEFDRLFR	18	52.0204
RGPHGWDTWLYTQ	13	35.7525
FHGAPARCPDPDHPTVACCQHGMPTFPHWHR	31	49.2979
LHHSNTDRIFAI	12	25.9371
LHHSNTDRIFAL	12	25.9371
LHHSNTDRLFAL	12	25.9371
DYAAYDPVFFLH	12	53.9808
NVESLSPLEGY	11	40.2011
DYSAFDPVFII	11	57.3601
DYSAFDPVFIL	11	57.3601
TIYDPIFFLH	10	52.2154

Peptide	Length	RT
TLYDPIFFLH	10	52.2154
SFHGAPARCPDPSHPTVACCVHGMPTFPHWHR	32	29.6204
FEIAHNLIHGL	11	38.5229
SYDPIFYIH	9	42.5061
SYDPLFYIH	9	42.5061
SYDPLFYLH	9	42.5061
FWSSYPFEGD	10	45.2653
LYVVQLEQALKEK	13	37.6517
KPLQPFQDK	9	14.8678
EFDGTPIDA	9	30.9591
PNFPHWH	7	22.9764
SLDYSAFDPVFFLH	14	54.3821
FHEPLHPF	8	26.6692
DYTLYDPIFFLHHS	14	51.5686
NTDRIWAVWQELQR	14	45.2098
LDSHLIKPPTVQ	12	29.0662
DLGHPFPDPTVIH	13	35.2696
AFDPVFIIVHS	11	51.3466
FDPVFIIVH	9	50.0717
DYSAFDPVF	9	50.4313
RGPHGWDTW	9	23.2607
ATFPHWHR	8	16.6183
SFHGAPARCPDPEHPTVACCQHGMPTFPH	29	27.3363
SLTSDVNPDPVTR	13	24.7536
IIERPSLIDDVFVV	14	51.7107
HSTIDRIWAI	10	35.8343
LEYTAYDPLFIIH	13	52.3839
LEYTAYDPLFLIH	13	52.3839
LEYTAYDPLFLLH	13	52.3839
YSAFDPFF	8	53.0639
GGETEMPFEFDRLFR	15	48.4412
WDWTKPNTK	9	21.9819
ALHSWLGGR	9	20.8221
DYSAFDPFFIIH	12	56.3088
LTGFDGEVL	9	42.1161
FEIAHNFIHAVVGGTELYSM	20	51.2915
GELIPGSAPGAN	12	28.8013
SLDYAAYDPVFFLHHT	16	51.0373
PADTFDYR	8	18.9227
KLAPPLVTL	9	40.1519
DSHLIKPPT	9	19.7093
VFKPGLPYT	9	32.2415
AYDPIFFIHH	10	40.0661

Peptide	Length	RT
AYDPIFFLHH	10	40.0661
AYDPLFFIHH	10	40.0661
AYDPLFFLHH	10	40.0661
QQDQGLGGFEAIAGF	15	51.8518
NHFHYEYDTL	10	24.7537
PNFPHWHR	8	17.9536
LTVDEALDKV	10	34.5492
GGELEMPFTFDRL	13	50.7609
GGPLEMPWSF	10	49.6021
QVEHALLEHGSSVAVPY	17	31.1709
LHGIGVSADVR	11	20.2008
LHGLGVSADVR	11	20.2008
LDPHILPEPSLIFVPG	16	54.9177
LYVEQVEHALL	11	44.6001
AIDAFDYDR	9	26.8006
FDYEYD	6	26.0456
WSSYPFEGD	9	37.2692
DEVIENPFAH	10	36.2379
DEVLENPFAH	10	36.2379
RYDDDYHF	8	20.5368
HLRPDSEY	8	10.3927
EYTSYDPI	8	31.8687
EYTSYDPL	8	31.8687
PSFPHWH	7	23.5739
VKQWEDSLTAH	11	20.9785
IQPEDVFDA	9	38.6315
SIPSGFEPDLS	11	39.1682
TIYDPIFF	8	54.441
TLYDPIFF	8	54.441
DYSAFDPF	8	48.6591
IYDPIFFLHH	10	46.3053
LYDPIFFLHH	10	46.3053
LIHNIGLSADVDIY	14	40.9638
LDYSAFDPVFFLH	13	54.4113
AIDELPSTF	9	41.4829
SLDYSAFDPVFF	12	56.1612
YSHPEDTF	8	17.9794
DSNPFPLTKR	10	22.9759
DYAAFDPV	8	39.0607
TISELNDILEER	12	40.6356
AAFLQWHRLYTVQFEDALR	19	45.7176
LRPDSEYHF	9	21.9549
LQLHWAQPY	9	36.5636

Peptide	Length	RT
HHNPFHDAE	9	6.8494
DLTDPLEDL	9	48.8566
VQFVPGVK	8	26.827
FDPNPFFSGR	10	37.5981
IHNIGLSADVE	11	27.2581
GGELEMPFTFD	11	51.9916
GFLSPLEPFK	10	45.6891
GGPLEMPWAFL	11	57.135
DYSAFDPVFFLH	12	54.5005
NYQYDDLNFHGL	12	39.873
SYSAFDPI	8	41.0732
GGPLEMPWVF	10	56.6578
SYSAFDPIFYIHHS	14	44.2363
SYSAFDPIFYLHHS	14	44.2363
HLRPDSEYHF	10	18.7664
TLEYTAYDPLFIIHHS	16	49.5201
TLEYTAYDPLFLLHHS	16	49.5201
AIDAFDYDRL	10	37.9559
YFEITKTVEKL	11	35.2416
YDVTNVFNKL	10	42.0873
KLKEPLKPF	9	19.8129
EYTAYDPLFIIHH	13	46.727
EYTAYDPLFLIHH	13	46.727
EYTAYDPLFLLHH	13	46.727
YHFVDHIT	8	22.5057
SVLGGELEMPFTFD	14	55.7129
TLDYSAFDPFFIIH	14	56.4555
YDDDYHFK	8	15.9531
AHGYIPSEDAY	11	24.2028
VTSDLTDPLEDL	12	46.8135
WQELQKIR	8	20.6415
WQELQKLR	8	20.6415
SVLGGELEMPFTF	13	56.6269
LTGFDGE	7	23.053
RLRFLLATLIR	11	29.0372
SAGEIESLRSAFLA	14	42.1149
NLESLSPGEVESLRAA	16	37.1056
QFAIWQALQK	10	43.9848
DYTLYDPIFFLHHSNVDR	18	47.7432
IHYLVGGHQTY	11	21.0824
QLPSDILPHPS	11	35.5912
IHHSTIDRIWAI	12	31.4927
TLQIEQALHR	10	25.5928

Peptide	Length	RT
IIERPSLIDDVFVVG	15	50.734
FFIGGSGTY	9	40.0657
QLPSDILPHP	10	38.1494
YDPIFYIH	8	43.0345
YDPIFYLH	8	43.0345
YDPLFYIH	8	43.0345
YDPLFYLH	8	43.0345
DYTAYDPIFFLHH	13	44.5204
GGELEMPFTFDRLYK	15	45.9117
FHGAPARCPDPEHPTVACCQHGMPTFPHWHR	31	33.3306
LSPLEPFSRDSNPFPLTK	18	42.0851
SAFDPFF	7	51.2334
LPSDILPHPS	10	33.516
FHGAPARCPDPDHPTVACCQHGMPTFPH	28	51.4011
LSPLEPFKR	9	27.7031
LTDPLEDL	8	42.194
SAFDPIFY	8	49.2979
NHFDYEYD	8	20.7419
SGMDRIWAI	9	41.2341
IHAVVGGTELYSMA	14	38.4099
PVFPHWHR	8	27.9631
EYTAYDPLFIIHHSNVDR	18	41.7289
EYTAYDPLFLIHHSNVDR	18	41.7289
EYTAYDPLFLLHHSNVDR	18	41.7289
GGAIEMPWAF	10	53.5013
GGALEMPWAF	10	53.5013
VIFVPAK	7	25.9645
TLDYSAFDPVFIIVHSGMDR	20	54.1836
HHNPFHDA	8	6.131
DYSAFDPVFF	10	55.8325
AFDPIFYIH	9	46.9797
AFDPIFYLH	9	46.9797
AFDPLFYLH	9	46.9797
LITVQAENALR	11	30.3299
LLTVQAENALR	11	30.3299
HWAQPY	6	18.5068
IIFVPGTK	8	31.7073
HHSNVDRQFAIWQAL	15	37.9434
FTTHLPNVPDLN	12	38.6574
DLGQLFPQPTVIH	13	48.6874
IIERPSLIDDVF	12	46.727
HYASYDPI	8	25.1741
HYASYDPL	8	25.1741

Peptide	Length	RT
DVTNVFNKL	9	38.5772
YSAYDPIFFIH	11	49.7106
YSAYDPIFFLH	11	49.7106
YSAYDPLFFIH	11	49.7106
AVFPHWHRLI	10	31.5199
AVFPHWHRLL	10	31.5199
QWEDSLTAH	9	24.0245
TTHLPNVPDLN	11	31.8423
WDWTLPIKELPA	12	52.667
LDPHILPEPS	10	34.052
VWAGFLLK	8	43.1451
YDVTNVFNK	9	29.8025
DDDYHFK	7	13.9066
DPVFIIVH	8	41.2088
FDPNPFFR	8	38.3027
DYSAFDPV	8	40.0053
FPDPTVIHE	9	31.2247
GGPLEMPW	8	48.6042
SYSAFDPIFYIHHSTIDRIWAI	22	52.5114
VVQIEQALKEK	11	23.2635
VVQLEQALKEK	11	23.2635
NGFQNIASFH	10	31.0895
YSRPIDTF	8	24.7521
EYSAYDPIFFIH	12	50.2652
EYSAYDPIFFLH	12	50.2652
EYSAYDPLFFIH	12	50.2652
IYDPIFFLH	9	51.485
LYDPIFFLH	9	51.485
HHSNVDRQFAIW	12	27.4405
EMPWTNE	7	29.8025
AYDPIFIIH	9	47.4366
AYDPLFIIH	9	47.4366
AYDPLFLIH	9	47.4366
AYDPLFLLH	9	47.4366
LFNDPEFGDESFF	13	53.7778
FHGDPGKCPTPTAKDRLSCCVHGMPTFPHWHR	32	31.9733
PGTDEVEE	8	44.0994
IVVQVEDALRR	11	28.6464
LVVQVEDALRR	11	28.6464
DNPFSSY	7	31.0113
GHPFPDPTVIH	11	25.8326
YDDDYHF	7	24.4151
YDPIFFIH	8	48.3032

Peptide	Length	RT
YDPIFFLH	8	48.3032
YDPLFFIH	8	48.3032
YDPLFFLH	8	48.3032
IPPFSYN	7	37.5441
TSYDPIFF	8	50.4038
TSYDPLFF	8	50.4038
PDPTVIHE	8	37.3512
AIHSWTGGQSPY	12	24.4654
DYSAFDPVFFL	11	57.6602
SYSAFDP	7	27.4674
VGPHGWDTWLYNQ	13	41.5918
VFAGFLLK	8	41.7018
HFVDHIVAV	9	29.0399
IGIPYWDWTR	10	47.9657
IGLPYWDWTR	10	47.9657
DYAAFDPVFFL	11	57.7168
YHFVDHIV	8	28.197
FDPIFYIH	8	48.8005
FDPIFYLH	8	48.8005
FDPLFYLH	8	48.8005
GAKIGIPYWDWTTAF	15	53.528
GAKLGIPYWDWTTAF	15	53.528
NVDALSPR	8	15.4245
RKPLPPFS	8	19.9167
HFVDHITA	8	18.4803
FDGTPIDA	8	28.2786
SLYDPIFF	8	54.529
EYTAYDPIF	9	46.3851
EYTAYDPLF	9	46.3851
LYVEQVEEALL	11	51.9621
GGPTESPW	8	25.9912
LDSHLIKPPT	10	25.2553
TTHLPNVPDLNLE	13	40.5562
GHVFPEPTVIHE	12	33.9416
FSLHNIGVSADVKVR	15	30.8585
EYSAYDPIFF	10	52.6388
EYSAYDPLFF	10	52.6388
DLGHPFPDPTVIHE	14	35.9481
DYSAFDPI	8	43.1451
DYSAFDPL	8	43.1451
FEGDWT	6	26.3068
SYNYPIE	7	26.8277
HGWDTW	6	28.0422

Peptide	Length	RT
LQQDQGLGGFEAIAGF	16	52.6668
EYTSYDPIFF	10	52.243
EYTSYDPLFF	10	52.243
GGELEMPFTFDR	12	44.6001
THLPNVPDLNLE	12	40.3077
GFLSPLEPFKR	11	38.7156
IGIPYWDWTT	10	53.6945
LGIPYWDWTT	10	53.6945
EMPWAYER	8	28.9322
GIPYWDWTKPNAA	13	41.5379
DYYDVWRDEVVPNPF	15	52.3551
HNPFHDA	7	8.2172
ASYDPIFYIH	10	43.5316
ASYDPLFYIH	10	43.5316
ASYDPLFYLH	10	43.5316
ILGGPLEMPWAF	12	57.0015
LLGGPLEMPWAF	12	57.0015
HHNPFHD	7	6.1589
TFDPNPF	7	37.8444
VEFEHVDTER	10	17.1253
WDWTSHVSELPK	12	39.8169
PNPFFSGR	8	25.5123
IENPFAH	7	29.8025
LENPFAH	7	29.8025
ESPWAY	6	30.3559
PVFIIVHS	8	36.7558
EMPWTN	6	28.3562
SDLTDPLEDL	10	45.6606
HNAIHYLVGGHQTY	14	20.8479
AFSPPYN	7	25.407
LQPGDYY	7	26.0456
FIIHHSNVDRQFAIW	15	37.0516
FLIHHSNVDRQFAIW	15	37.0516
FLLHHSNVDRQFAIW	15	37.0516
KIGIPYWDWTT	11	48.8005
KLGIPYWDWTT	11	48.8005
YVEQVEHALLEHGSAVSVPY	20	41.5093
WEDSLTAH	8	21.428
ALPPLVTY	8	44.1514
DGSFNEF	7	30.883
QFAIWQAL	8	53.1799
PNFPHW	6	31.5477
YSHPEDTFAY	10	25.2488

Peptide	Length	RT
QRFDPNPFFSG	11	38.0365
EVIENPFAH	9	33.5144
EVLENPFAH	9	33.5144
FDPNPFF	7	49.4134
SYNYPIEN	8	24.4151
LLDPHILPD	9	39.3285
YHFVDHIVA	9	27.2581
FHYDYDNLE	9	27.9631
YASYDPIFYIH	11	46.0264
YASYDPLFYIH	11	46.0264
YASYDPLFYLH	11	46.0264
VNPDPVTR	8	12.7035
VVPNPFAH	8	29.6213
GHPFPDPTVIHE	12	26.9648
FRLHGIGVSA	10	27.571
FRLHGLGVSA	10	27.571
WDWTKPN	7	27.1256
SFGPPYN	7	26.0456
FHYEYDNL	8	28.9291
LYELTLDAL	9	51.7664
FSFGPPYN	8	38.795
KIGIPYWDWTTAF	13	53.585
KLGIPYWDWTTAF	13	53.585
HGSPLGIPY	9	32.0259
VFAGFILH	8	42.3931
VFAGFLIH	8	42.3931
VFAGFLLH	8	42.3931
SILGGETEMPFEFDRLFRI	19	54.8279
IHNIGLSADVDIY	13	37.1877
EGHEIACCIHGMPVFPHWHR	20	29.3873
SSLEYTVYDPIFFLHH	16	52.5017
TVYDPIFF	8	53.3296
HLRPDSEYHFDV	12	21.5594
LLVVQVEDALRRRGSPIGVPY	21	45.8575
YSAYDPIFFIHH	12	43.3647
YSAYDPIFFLHH	12	43.3647
YSAYDPLFFIHH	12	43.3647
WTGGQSPY	8	21.428
VIENPFAH	8	29.8025
VLENPFAH	8	29.8025
YHFVDHITAV	10	29.1434
TVQFEDALR	9	28.595
SPLEPFKR	8	38.7156

Peptide	Length	RT
LSPLEPFSR	9	31.3573
VQFEDALR	8	26.8006
NHFDYEY	7	21.9027
YYDPWR	6	25.7797
IYIVVEDH	8	31.7089
AYDPIFFIH	9	47.3596
AYDPIFFLH	9	47.3596
AYDPLFFIH	9	47.3596
AYDPLFFLH	9	47.3596
GMPTFPH	7	26.0723
AFDPIFY	7	48.5766
AFDPLFY	7	48.5766
RVHFFIGGS	9	28.3042
EMPWAY	6	38.8972
WDWTLPIK	8	47.8266
TAYDPIF	7	41.2208
TAYDPLF	7	41.2208
LSPLEPFK	8	33.8657
IYDPIFF	7	54.4665
LYDPIFF	7	54.4665
GGPTESPWAYDR	12	25.4888
FLSPLEPF	8	53.0321
LHWAQPY	7	27.4148
AIEMPWA	7	45.045
ALEMPWA	7	45.045
PAGEFYIL	8	48.6874
PAGEFYLL	8	48.6874
VFKPGLPY	8	31.9218
IGGSGTY	7	12.9412
PSDILPHPS	9	47.8796
EEHFHY	6	12.5404
APREQLFNDPEFGSESFFYRQ	21	45.8489
YHFVDHITA	9	24.4416
WSPWAG	6	39.6513
ATFPQWHRLY	10	32.6662
QWEDALTAHGAK	12	22.3485
VAGEDAVTTR	10	11.5414
LGHVFPEPTVIHE	13	37.8468
NNPFNHG	7	10.2297
GGPLEMPWS	9	42.1796
EFEHVDTER	9	12.0009
GGEKEMPWAYER	12	23.9727
DNLDIGGH	8	17.3415

Peptide	Length	RT
DNLDLGGH	8	17.3415
GVPYWDW	7	48.9405
YTDEVVIVER	10	28.1469
EIDDYINT	8	30.2
ELDDYLNT	8	30.2
PDDDYHF	7	27.9114
SDGSFSAF	8	32.0281
GGELEMPFTF	10	53.528
RDPQPQLFNNEYFYEQ	16	40.1482
SYPFEGD	7	26.6935
FHGYPARCEHDNHPVACCLHGM	22	23.8694
FPHWHR	6	13.6627
NPFPITR	7	39.1153
FVDKIWVV	8	43.4214
VGPHGWD	7	16.8814
DEVIENPF	8	40.7441
DEVLENPF	8	40.7441
LRPDSEYH	8	9.1138
FHGAPAR	7	5.5202
HGAPARCPDPDHPTVACCQHGMPTFPH	27	49.5475
VVGAGPSGTY	10	19.8638
DNSADGF	7	15.4495
LNEPLHPF	8	31.8423
IIERPSLIDDVFV	13	49.2434
DSNPFPLTK	9	28.5139
NEPLHPF	7	25.3814
WDWTQPIT	8	46.8951
WDWTQPLT	8	46.8951
GGETEMPFEFDRLF	14	52.6388
GGSLEMPWS	9	41.1566
YSAYDPIFF	9	52.3275
YSAYDPLFF	9	52.3275
FILHGIQ	7	31.6015
FILHGLQ	7	31.6015
FLLHGIQ	7	31.6015
AYDPIFFI	8	56.3673
AYDPIFFL	8	56.3673
AYDPLFFI	8	56.3673
AYDPLFFL	8	56.3673
ENPFAH	6	29.8025
WGDHTE	6	6.8494
HLRPDSEYHFDVH	13	18.0834
LFNSVLEAL	9	52.9193

Peptide	Length	RT
YPFEGD	6	24.7537
TQSPEWGDH	9	15.744
GTQLPSDILPHPS	13	36.6456
VGPHGW	6	16.7538
AIPYWDWTSHVSELPK	16	48.7694
LGHVFPEPTVIHEV	14	41.9479
VPYWDW	6	47.7158
FGPPYN	6	23.3911
SFHGAPARCPDPDHPKVACCVHGMPTFPHWHR	32	33.462
LHIDDPLVTQR	11	29.7761
LYTAFDPIFYLH	12	54.1553
LYTAFDPLFYLH	12	54.1553
FNGLTIPQLAR	11	41.3434
LLAALPRPL	9	55.5575
LPVFPH	6	28.9322
EGCTCYGQ	8	8.6398
EENNPFHH	8	11.2942
LPNVPDLN	8	35.4304
LIVPKGTHIR	10	17.9569
GFNLNKFTR	9	25.3814
VHFFIG	6	35.0514
SAFDPIF	7	47.4902
SAFDPLF	7	47.4902
LIPGSAPGANR	11	17.2378
RPDSEYH	7	9.1123
HSNVDRQFAIW	11	32.2151
SKEMPWTN	8	21.8768
LPNVPDLNLE	10	45.8016
WDWTAPVR	8	35.7
GLPFPNLPY	9	52.4427
EEPLHPF	7	27.3632
KLTGFDGE	8	18.3213
SLLYTGYDPIFYLH	14	52.6098
HGMPTFPH	8	20.0445
HEPLHPF	7	18.8977
SLSPGEVESLRAA	13	29.3555
HGSSVAIPY	9	25.2805
HGSSVALPY	9	25.2805
VFKPGLP	7	28.068
PVFIIVH	7	36.7544
HLPNVPDLN	9	29.8823
TIPQLENLLK	10	43.9314
SYSLSRATSCTSCPAGFSCATTNSAPT	27	33.4902

Peptide	Length	RT
FILHGLQST	9	31.5459
LYTVQFEDALK	11	39.0484
SAYDPIFF	8	50.7078
SAYDPLFF	8	50.7078
SAYDPIF	7	39.5723
SAYDPLF	7	39.5723
GQLFPQP	7	34.6554
IHAVVGGTELYSM	13	37.7658
YDDDYH	6	7.6538
IVVQVEDALR	10	33.1439
LVVQVEDALR	10	33.1439
IGIPYWDWTTAFTALPS	17	57.3037
INEPLKPF	8	31.3048
LNEPLKPF	8	31.3048
MPTFPH	6	24.4928
YEYDNLEFNGL	11	46.5495
NILGGPTESPWAYDR	15	39.2472
WEQLQKLR	8	20.6415
TLSAGEVESLRSAFLAIQ	18	51.6251
YTAYDPI	7	33.3306
YTAYDPL	7	33.3306
FFGDSS	6	22.3219
FKPGLPY	7	28.9863
SILGGETEMPFE	12	47.0634
HIDDVNQN	8	6.049
FIIVHSGMDR	10	23.7595
HAGDFF	6	24.4143
DYRNHFHYEYDTL	13	27.4427
IIILVH	6	32.2132
IILIVH	6	32.2132
ILIIVH	6	32.2132
ILILVH	6	32.2132
ILLLVH	6	32.2132
LILLVH	6	32.2132
LLILVH	6	32.2132
LLLIVH	6	32.2132
LLLLVH	6	32.2132
LQADNTNDGWANLASFH	17	34.7352
FHGAPFL	7	34.0336
LIHNIGLSAD	10	28.0422
LLHNIGLSAD	10	28.0422
KRGPHGWDTW	10	17.8504
HSNTDRIFAI	10	30.2508

Peptide	Length	RT
HSNTDRIFAL	10	30.2508
HSNTDRLFAL	10	30.2508
VHTPLKPF	8	23.0015
YHFVDH	6	12.7035
SLEYSAYDPIFF	12	54.4712
SLEYSAYDPLFF	12	54.4712
GSHIGIPY	8	27.0717
GSHLGIPY	8	27.0717
EHAVPDTLF	9	28.5684
FLQWHR	6	27.3632

Table S3 Peptides with potential bioactivity identified in WJSS

Peptide	Hydrophobicity (kcal/mol)	pI	Charge at pH7	Peptide Ranker	ToxinPred
FDPNPFF	0.05	3.8	-1	0.987722	Non-Toxin
SAFDPFF	0.15	3.8	-1	0.984876	Non-Toxin
FDPNPFFR	-0.18	6.19	0	0.983403	Non-Toxin
YSAFDPFF	0.13	3.8	-1	0.970804	Non-Toxin
FDPNPFFSGR	-0.15	6.19	0	0.966175	Non-Toxin
AYDPLFFL	0.22	3.8	-1	0.96568	Non-Toxin
PNFPHW	-0.03	7.1	0.5	0.964767	Non-Toxin
SAFDPLF	0.14	3.8	-1	0.962871	Non-Toxin
GGPLEMPWAFL	0.19	4	-1	0.962729	Non-Toxin
WSPWAG	0.14	5.88	0	0.962269	Non-Toxin
AYDPIFFL	0.24	3.8	-1	0.960478	Non-Toxin
AFDPLFY	0.18	3.8	-1	0.959079	Non-Toxin
FHGAPFL	0.24	7.1	0.5	0.956122	Non-Toxin
GGPLEMPWAF	0.16	4	-1	0.954577	Non-Toxin
SAYDPLFF	0.12	3.8	-1	0.953924	Non-Toxin
LYDPIFF	0.24	3.8	-1	0.953342	Non-Toxin
DYSAFDPFF	0.04	3.57	-2	0.950402	Non-Toxin
FDPNPFFSGK	-0.09	6.19	0	0.948018	Non-Toxin
AYDPLFFI	0.24	3.8	-1	0.94659	Non-Toxin
SLYDPIFF	0.18	3.8	-1	0.944681	Non-Toxin
FPHWHR	-0.27	10.11	2	0.944365	Non-Toxin
IYDPIFF	0.27	3.8	-1	0.942301	Non-Toxin
AFDPIFY	0.2	3.8	-1	0.941688	Non-Toxin
PNPFFSGR	-0.18	10.11	1	0.940978	Non-Toxin
SAYDPIFF	0.15	3.8	-1	0.940947	Non-Toxin
GGPLEMPWVF	0.19	4	-1	0.937993	Non-Toxin
SAFDPIF	0.16	3.8	-1	0.937929	Non-Toxin
AFDPLFYLH	0.15	5.09	-0.5	0.936129	Non-Toxin
PSFPHWH	-0.03	7.26	1	0.935857	Non-Toxin
PSFPHWHR	-0.25	10.11	2	0.935517	Non-Toxin
FDPLFYLH	0.14	5.09	-0.5	0.934909	Non-Toxin
AYDPIFFI	0.27	3.8	-1	0.934897	Non-Toxin
AYDPLFFLH	0.15	5.09	-0.5	0.933114	Non-Toxin
AVFPHWHRLL	0.02	10.11	2	0.928971	Non-Toxin
PNFPHWHR	-0.3	10.11	2	0.928894	Non-Toxin
HAGDFF	0.09	5.09	-0.5	0.928592	Non-Toxin
PNFPHWH	-0.09	7.26	1	0.928509	Non-Toxin
DYAAFDPVFFL	0.17	3.57	-2	0.928113	Non-Toxin
RFDPNPFFSGR	-0.3	9.95	1	0.928046	Non-Toxin
GGPLEMPWSF	0.11	4	-1	0.927581	Non-Toxin

Peptide	Hydrophobicity (kcal/mol)	pI	Charge at pH7	Peptide Ranker	ToxinPred
VPYWDW	0.09	3.8	-1	0.926596	Non-Toxin
YSAYDPLFF	0.11	3.8	-1	0.925631	Non-Toxin
AYDPIFFLH	0.17	5.09	-0.5	0.925573	Non-Toxin
FDPIFYLH	0.16	5.09	-0.5	0.924561	Non-Toxin
YDPLFFLH	0.14	5.09	-0.5	0.924076	Non-Toxin
SAFDPIFY	0.15	3.8	-1	0.923427	Non-Toxin
GGALEMPWAF	0.19	4	-1	0.922301	Non-Toxin
GVPYWDW	0.1	3.8	-1	0.921207	Non-Toxin
YDPIFFLH	0.16	5.09	-0.5	0.920659	Non-Toxin
AFDPIFYLH	0.17	5.09	-0.5	0.919865	Non-Toxin
GGPLEMPW	0.09	4	-1	0.916002	Non-Toxin
FGPPYN	0	5.88	0	0.915161	Non-Toxin
DYSAFDPVFFL	0.13	3.57	-2	0.912603	Non-Toxin
YSAYDPIFF	0.13	3.8	-1	0.90532	Non-Toxin
FSFGPPYN	0.04	5.88	0	0.904396	Non-Toxin
GGAIEMPWAF	0.21	4	-1	0.902522	Non-Toxin
ILGGPLEMPWAF	0.24	4	-1	0.901312	Non-Toxin
AYDPLFFIH	0.17	5.09	-0.5	0.898639	Non-Toxin
GGPLEMPWSFDRLFKY	-0.08	6.42	0	0.898112	Non-Toxin
YDPLFFIH	0.16	5.09	-0.5	0.89749	Non-Toxin
LYDPIFFLH	0.2	5.09	-0.5	0.896249	Non-Toxin
TSYDPLFF	0.07	3.8	-1	0.894552	Non-Toxin
SAYDPLF	0.05	3.8	-1	0.894005	Non-Toxin
LLGGPLEMPWAF	0.22	4	-1	0.893586	Non-Toxin
DYSAFDPVFF	0.09	3.57	-2	0.892513	Non-Toxin
NEPLKPFSFEGFNLNKF TR	-0.21	8.93	1	0.891138	Non-Toxin
RFDPNPFFSGK	-0.24	9.1	1	0.890258	Non-Toxin
IGIPYWDWTR	-0.04	6.19	0	0.889393	Non-Toxin
AVFPHWHRLI	0.04	10.11	2	0.887098	Non-Toxin
QRFDPNPFFSG	-0.2	6.19	0	0.886884	Non-Toxin
YYDPWR	-0.36	6.18	0	0.884821	Non-Toxin
IYDPIFFLH	0.23	5.09	-0.5	0.884329	Non-Toxin
YDPIFFIH	0.19	5.09	-0.5	0.88395	Non-Toxin
FDPIFYIH	0.19	5.09	-0.5	0.883682	Non-Toxin
AIPYWDWTLPIK	0.07	6.18	0	0.883618	Non-Toxin
AYDPIFFIH	0.2	5.09	-0.5	0.879543	Non-Toxin
WDWTLPIK	-0.01	6.19	0	0.879171	Non-Toxin
IGLPYWDWTR	-0.06	6.19	0	0.879048	Non-Toxin
QQRFDPNPFFSGR	-0.36	9.95	1	0.878625	Non-Toxin
SFGPPYN	-0.04	5.88	0	0.876404	Non-Toxin
ALPYWDWTLPIK	0.05	6.18	0	0.875085	Non-Toxin

Peptide	Hydrophobicity (kcal/mol)	pI	Charge at pH7	Peptide Ranker	ToxinPred
HGWDTW	-0.07	5.09	-0.5	0.874009	Non-Toxin
FLQWHR	-0.22	10.11	1.5	0.872833	Non-Toxin
DYSAFDPF	-0.03	3.57	-2	0.872588	Non-Toxin
QRFDPNPFFSGK	-0.28	9.1	1	0.872366	Non-Toxin
PVFPHWHR	-0.15	10.11	2	0.870215	Non-Toxin
SLDYAAFDPVFFL	0.17	3.57	-2	0.867676	Non-Toxin
LGLPFPNLPYL	0.19	5.88	0	0.867324	Non-Toxin
AFDPIFYIH	0.2	5.09	-0.5	0.866123	Non-Toxin
FHEPLHPF	0.02	6.01	0	0.865769	Non-Toxin
EGHEIACCIHGMPVFPHWHR	-0.04	6.46	1	0.864592	Toxin
TFDPNPF	-0.07	3.8	-1	0.861542	Non-Toxin
VWAGFLLK	0.24	9.11	1	0.861315	Non-Toxin
TLYDPIFF	0.19	3.8	-1	0.860734	Non-Toxin
FLSPLEPF	0.16	4	-1	0.859468	Non-Toxin
ALHSWLGGR	-0.05	10.11	1.5	0.859212	Non-Toxin
KIGIPYWDWTTAF	0.08	6.18	0	0.858618	Non-Toxin
TSYDPIFF	0.09	3.8	-1	0.855338	Non-Toxin
QQDQGLGGFEAIAGFHGAPF	0.04	4.36	-1.5	0.854701	Non-Toxin
DYAAFDPVFFLH	0.13	4.2	-1.5	0.853075	Non-Toxin
GAKLGIPYWDWTTAF	0.08	6.18	0	0.852735	Non-Toxin
GAKIGIPYWDWTTAF	0.09	6.18	0	0.851772	Non-Toxin
AVFPHWHR	-0.11	10.11	2	0.851768	Non-Toxin
AYDPLFFLHH	0.1	5.99	0	0.849934	Non-Toxin
KLGIPYWDWTTAF	0.06	6.18	0	0.849342	Non-Toxin
PAGEFYLL	0.18	4	-1	0.847959	Non-Toxin
GFLSPLEPFKR	-0.13	9.1	1	0.847401	Non-Toxin
AYDPLFLLH	0.14	5.09	-0.5	0.846841	Non-Toxin
GLPFPNLPY	0.11	5.88	0	0.845986	Non-Toxin
YSAYDPIFFLH	0.12	5.09	-0.5	0.845154	Non-Toxin
SAYDPIF	0.08	3.8	-1	0.843567	Non-Toxin
TIYDPIFF	0.22	3.8	-1	0.841755	Non-Toxin
YTSYDPLFF	0.06	3.8	-1	0.840455	Non-Toxin
AFSPPYN	-0.02	5.88	0	0.836953	Non-Toxin
AYDPIFFLHH	0.12	5.99	0	0.835832	Non-Toxin
SLDYAAFDPVFF	0.14	3.57	-2	0.834235	Non-Toxin
YDPLFYLH	0.07	5.09	-0.5	0.831616	Non-Toxin
SYDPLFYLH	0.03	5.09	-0.5	0.830888	Non-Toxin
DYSAFDPVFFLH	0.08	4.2	-1.5	0.830319	Non-Toxin
WDWTQPLT	-0.07	3.8	-1	0.827988	Non-Toxin

Peptide	Hydrophobicity (kcal/mol)	pI	Charge at pH7	Peptide Ranker	ToxinPred
VGVPYWDWTAPVRSLP SLF	0.06	6.19	0	0.823507	Non-Toxin
IGIPYWDWTT	0.12	3.8	-1	0.821818	Non-Toxin
EYSAYDPLFF	0.04	3.67	-2	0.821165	Non-Toxin
QFAIWQALQKFR	-0.13	11.01	2	0.819803	Non-Toxin
WDWTAPVR	-0.15	6.19	0	0.819539	Non-Toxin
ATFPHWHR	-0.2	10.11	2	0.815692	Non-Toxin
VFAGFLLK	0.27	9.11	1	0.815684	Non-Toxin
DYSAFDPFFIIH	0.12	4.2	-1.5	0.814438	Non-Toxin
RGPHGWDTW	-0.23	7.1	0.5	0.81254	Non-Toxin
YDPIFYLH	0.09	5.09	-0.5	0.811657	Non-Toxin
DYSAFDPVFIL	0.14	3.57	-2	0.810533	Non-Toxin
GFLSPLEPFK	0.03	6.35	0	0.810408	Non-Toxin
SYSAFDPIFY	0.09	3.8	-1	0.806216	Non-Toxin
ASYDPLFYLH	0.05	5.09	-0.5	0.804229	Non-Toxin
AILGGPLEMPWSFDR	0.01	4.38	-1	0.803764	Non-Toxin
AYDPLFFIHH	0.12	5.99	0	0.801588	Non-Toxin
NPFPITR	-0.2	10.11	1	0.800349	Non-Toxin
WDWTKPN	-0.28	6.19	0	0.800321	Non-Toxin
YSAYDPLFFIH	0.12	5.09	-0.5	0.798451	Non-Toxin
LGIPYWDWTT	0.1	3.8	-1	0.798027	Non-Toxin
SLDYSAFDPVFF	0.1	3.57	-2	0.796936	Non-Toxin
TAYDPLF	0.06	3.8	-1	0.794268	Non-Toxin
DYSAFDPVF	0.03	3.57	-2	0.79421	Non-Toxin
GGPLEMPWS	0.05	4	-1	0.793125	Non-Toxin
VHFFIG	0.38	7.1	0.5	0.791058	Non-Toxin
LDPHILPEPSLIFVPG	0.13	4.36	-1.5	0.79028	Non-Toxin
YTSYDPIFF	0.08	3.8	-1	0.78882	Non-Toxin
LRKPLPPFSL	-0.11	11.01	2	0.788648	Non-Toxin
EYSAYDPIFF	0.06	3.67	-2	0.785651	Non-Toxin
SYDPLFYIH	0.05	5.09	-0.5	0.783023	Non-Toxin
VFAGFLLH	0.35	7.1	0.5	0.781811	Non-Toxin
YDPLFYIH	0.09	5.09	-0.5	0.781745	Non-Toxin
AYDPIFFIHH	0.14	5.99	0	0.780583	Non-Toxin
GGELEMPFTF	0.08	3.8	-2	0.778836	Non-Toxin
TVYDPIFF	0.19	3.8	-1	0.778824	Non-Toxin
LYDPIFFLHH	0.14	5.99	0	0.778451	Non-Toxin
AYDPLFLIH	0.16	5.09	-0.5	0.777851	Non-Toxin
GMPTFPH	0.04	7.1	0.5	0.777304	Non-Toxin
SFEGFNLNKFTR	-0.22	9.1	1	0.77676	Non-Toxin
PDDDYHF	-0.29	3.93	-2.5	0.77332	Non-Toxin
GGELEMPFTFDRL	-0.09	4.14	-2	0.772395	Non-Toxin

Peptide	Hydrophobicity (kcal/mol)	pI	Charge at pH7	Peptide Ranker	ToxinPred
YSAYDPIFFIH	0.14	5.09	-0.5	0.770686	Non-Toxin
SLDYAAFDPVFFLH	0.13	4.2	-1.5	0.768644	Non-Toxin
MPTFPH	0.03	7.1	0.5	0.768547	Non-Toxin
FKPGLPY	0.01	8.94	1	0.767469	Non-Toxin
SPLEPFKR	-0.34	9.1	1	0.766197	Non-Toxin
AYDPLFIIH	0.19	5.09	-0.5	0.765659	Non-Toxin
HEPLHPF	-0.06	6.01	0	0.765057	Non-Toxin
KIGIPYWDWTT	0.01	6.18	0	0.763831	Non-Toxin
QFAIWQAL	0.17	5.88	0	0.763221	Non-Toxin
VGPHGW	0.13	7.1	0.5	0.760986	Non-Toxin
SYSAFDPIFYLH	0.09	5.09	-0.5	0.76044	Non-Toxin
ASYDPLFYIH	0.07	5.09	-0.5	0.758706	Non-Toxin
DYSAFDPL	-0.04	3.57	-2	0.757184	Non-Toxin
PAGEFYIL	0.2	4	-1	0.756848	Non-Toxin
HNALHSWLGGR	-0.13	10.11	2	0.755847	Non-Toxin
FWSSYPFEGD	-0.02	3.67	-2	0.751612	Non-Toxin
LLAALPRPL	0.08	10.11	1	0.751554	Non-Toxin
VGVPYWDWTAPVR	0	6.19	0	0.751295	Non-Toxin
YDPIFYIH	0.11	5.09	-0.5	0.750983	Non-Toxin
IYDPIFFLHH	0.16	5.99	0	0.750593	Non-Toxin
WDWTQPIT	-0.05	3.8	-1	0.749262	Non-Toxin
TLYDPIFFLH	0.17	5.09	-0.5	0.74886	Non-Toxin
GQLFPQP	-0.03	5.88	0	0.746893	Non-Toxin
VFAGFLLKGIK	0.17	10.02	2	0.746655	Non-Toxin
NEPLHPF	-0.09	5.25	-0.5	0.745839	Non-Toxin
LDYSAFDPVFFLH	0.12	4.2	-1.5	0.744792	Non-Toxin
DYAAYDPVFFLH	0.08	4.2	-1.5	0.743283	Non-Toxin
SDGSFSAF	0.02	3.8	-1	0.740404	Non-Toxin
FHGDPGKCPTPTAKDR LSCCVHGMPTFPHWHR	-0.19	8.77	4	0.739621	Non-Toxin
LFNDPEFGSESFFYR	-0.12	4.14	-2	0.738993	Non-Toxin
SYDPIFYIH	0.07	5.09	-0.5	0.738484	Non-Toxin
KLGIPYWDWTT	-0.01	6.18	0	0.732133	Non-Toxin
LSPLEPFKR	-0.25	9.1	1	0.7308	Non-Toxin
GGETEMPFEFDRLF	-0.12	4.01	-3	0.729607	Non-Toxin
HGMPTFPH	-0.01	7.26	1	0.728434	Non-Toxin
ALEMPWA	0.14	4	-1	0.725629	Non-Toxin
QQDQGLGGFEAIAGF	0.01	3.67	-2	0.725333	Non-Toxin
AYDPIFIIH	0.21	5.09	-0.5	0.724117	Non-Toxin
EMPWAY	0.03	4	-1	0.721443	Non-Toxin
TIYDPIFFLH	0.19	5.09	-0.5	0.720927	Non-Toxin
YSAYDPIFFLHH	0.08	5.99	0	0.720699	Non-Toxin

Peptide	Hydrophobicity (kcal/mol)	pI	Charge at pH7	Peptide Ranker	ToxinPred
AIDAFDYDRL	-0.15	3.94	-2	0.719302	Non-Toxin
GFNLNKFTR	-0.27	11.01	2	0.719197	Non-Toxin
SLDYSAFDPVFFLH	0.09	4.2	-1.5	0.718897	Non-Toxin
YASYDPLFYLH	0.05	5.09	-0.5	0.71876	Non-Toxin
GIPYWDWTKPNAA	-0.05	6.18	0	0.717602	Non-Toxin
LFNDPEFGDESFFYR	-0.15	3.92	-3	0.715791	Non-Toxin
KRGPHGWDTW	-0.32	9.1	1.5	0.715358	Non-Toxin
HWAQPY	-0.09	7.09	0.5	0.706698	Non-Toxin
WSSYPFEGD	-0.09	3.67	-2	0.705848	Non-Toxin
SGMDRIWAI	-0.03	6.19	0	0.704967	Non-Toxin
DSNPFPLTK	-0.21	6.19	0	0.704635	Non-Toxin
EYSAYDPIFFLH	0.06	4.36	-1.5	0.704505	Non-Toxin
VFKPGLP	0.09	9.11	1	0.704029	Non-Toxin
GGSLEMPWS	0.03	4	-1	0.700972	Non-Toxin
ATFPQWHRLY	-0.13	9.1	1.5	0.700165	Non-Toxin
SYSLSRATSCTSCPAGF SCATTNSAPT	-0.1	8.26	1	0.699838	Non-Toxin
AIEMPWA	0.17	4	-1	0.698609	Non-Toxin
SYSAFDPIFYIH	0.1	5.09	-0.5	0.696194	Non-Toxin
SLEYSAYDPLFF	0.05	3.67	-2	0.694672	Non-Toxin
TAYDPIF	0.09	3.8	-1	0.693754	Non-Toxin
ASYDPIFYIH	0.09	5.09	-0.5	0.692954	Non-Toxin
FHGAPAR	-0.14	10.11	1.5	0.69234	Non-Toxin
EYTSYDPLFF	-0.01	3.67	-2	0.692168	Non-Toxin
DSNPFPLTKR	-0.37	9.1	1	0.691855	Non-Toxin
GGETEMPFEFDRLFR	-0.23	4.41	-2	0.689687	Non-Toxin
DYSAFDPVFII	0.16	3.57	-2	0.689253	Non-Toxin
YSAYDPLFFIHH	0.08	5.99	0	0.689067	Non-Toxin
PADTFDYR	-0.32	4.21	-1	0.686628	Non-Toxin
RKPLPPFS	-0.27	11.01	2	0.682755	Non-Toxin
FEIAHNFIHAL	0.15	6.01	0	0.681248	Non-Toxin
DGSFNEF	-0.12	3.67	-2	0.67999	Non-Toxin
VFAGFILH	0.38	7.1	0.5	0.678388	Non-Toxin
EYTAYDPLFLL	0.08	3.67	-2	0.677886	Non-Toxin
INEPLKPF	-0.08	6.35	0	0.67661	Non-Toxin
HGSPLGIPY	0.09	7.09	0.5	0.676371	Non-Toxin
FDPVFIIVH	0.29	5.09	-0.5	0.675648	Non-Toxin
GGETEMPFEFDRLFRI	-0.17	4.41	-2	0.674511	Non-Toxin
FEGDWT	-0.06	3.67	-2	0.67365	Non-Toxin
YASYDPLFYIH	0.07	5.09	-0.5	0.671695	Non-Toxin
QLPSDILPHP	-0.05	5.09	-0.5	0.671514	Non-Toxin
AFDPVFIIVH	0.28	5.09	-0.5	0.669823	Non-Toxin

Peptide	Hydrophobicity (kcal/mol)	pI	Charge at pH7	Peptide Ranker	ToxinPred
NDPEFGDESFFYR	-0.26	3.92	-3	0.669511	Non-Toxin
IPPFSYN	0.05	5.88	0	0.668123	Non-Toxin
YDDDYHF	-0.27	3.93	-2.5	0.667197	Non-Toxin
LNEPLKPF	-0.1	6.35	0	0.665633	Non-Toxin
LSPLEPFSR	-0.15	6.36	0	0.665514	Non-Toxin
SLEYSAYDPIFF	0.07	3.67	-2	0.661787	Non-Toxin
TAYDPLFYLH	0.06	5.09	-0.5	0.661029	Non-Toxin
LFNDPEFGDESFF	-0.04	3.44	-4	0.658534	Non-Toxin
LHWAQPY	0	7.09	0.5	0.658024	Non-Toxin
LPVFPH	0.19	7.1	0.5	0.655467	Non-Toxin
LQQDQGLGGFEAIAGF	0.05	3.67	-2	0.653725	Non-Toxin
YSAYDPIFFIHH	0.09	5.99	0	0.653143	Non-Toxin
LYTAFDPLFYLH	0.14	5.09	-0.5	0.649947	Non-Toxin
SYSAFDP	-0.06	3.8	-1	0.649555	Non-Toxin
DYTLYDPIFFLH	0.08	4.2	-1.5	0.648654	Non-Toxin
VFAGFLIH	0.38	7.1	0.5	0.648001	Non-Toxin
NNPFNHG	-0.23	7.1	0.5	0.643815	Non-Toxin
VGPHGWDTWLYNQ	-0.04	5.09	-0.5	0.643724	Non-Toxin
GHPFPDPTVIH	0.01	5.99	0	0.643131	Non-Toxin
SYSAFDPI	0.04	3.8	-1	0.642616	Non-Toxin
FRYDDDYHFK	-0.42	5.31	-0.5	0.641782	Non-Toxin
LSPLEPFK	-0.06	6.35	0	0.640727	Non-Toxin
SSLDYTLYDPIFFLH	0.06	4.2	-1.5	0.640107	Non-Toxin
DYSAFDPVFFLHHA	0.06	5.06	-1	0.640088	Non-Toxin
LNEPLHPF	-0.02	5.25	-0.5	0.63905	Non-Toxin
WDWTKPNTK	-0.36	8.94	1	0.637434	Non-Toxin
ESPWAY	-0.05	4	-1	0.635533	Non-Toxin
EYSAYDPLFFIH	0.06	4.36	-1.5	0.633192	Non-Toxin
RGPHGWDTWLYTQ	-0.18	7.09	0.5	0.631463	Non-Toxin
SVLGGELEMPFTFDRL	-0.02	4.14	-2	0.631341	Non-Toxin
LFNDPEFGDESFFY	-0.04	3.44	-4	0.629525	Non-Toxin
SLDYTLYDPIFFLH	0.09	4.2	-1.5	0.628399	Non-Toxin
DYSAFDPVFIIVHSGMDR	-0.03	4.42	-1.5	0.628027	Non-Toxin
LRKPLPPFS	-0.18	11.01	2	0.627826	Non-Toxin
DDDYHFK	-0.43	4.42	-1.5	0.627145	Non-Toxin
GSSKEMPWTNEILFKY	-0.14	6.49	0	0.627046	Non-Toxin
LSSLDYSAFDPVFFLH	0.1	4.2	-1.5	0.626946	Non-Toxin
LSPLEPFSRDSNPFPLTK	-0.18	6.42	0	0.626667	Non-Toxin
RVHFFIGGS	0.04	10.11	1.5	0.626609	Non-Toxin
ISSLDYSAFDPVFFLH	0.11	4.2	-1.5	0.624901	Non-Toxin
SISSLDYSAFDPVFFLH	0.09	4.2	-1.5	0.624888	Non-Toxin

Peptide	Hydrophobicity (kcal/mol)	pI	Charge at pH7	Peptide Ranker	ToxinPred
YDDDYHFH	-0.29	4.4	-2	0.623278	Non-Toxin
EYTSYDPIFF	0.01	3.67	-2	0.623078	Non-Toxin
GGELEMPFTFDRLYK	-0.15	4.68	-1	0.619771	Non-Toxin
LFNDPEFGDESFFYRQ	-0.18	3.92	-3	0.619473	Non-Toxin
GSHLGIPY	0.11	7.09	0.5	0.616368	Non-Toxin
EYSAYDPIFFIH	0.07	4.36	-1.5	0.616063	Non-Toxin
GGELEMPFTFDR	-0.14	4.14	-2	0.611123	Non-Toxin
SLSSLDYSAFDPVFFLH	0.08	4.2	-1.5	0.611058	Non-Toxin
DYSAFDPI	-0.02	3.57	-2	0.610237	Non-Toxin
EEPLHPF	-0.09	4.51	-1.5	0.608481	Non-Toxin
HSNVDRQFAIWQALQKFR	-0.27	10.84	2.5	0.608325	Non-Toxin
FEIAHNLIHGL	0.13	6.01	0	0.608178	Non-Toxin
SLSYSAFDPIFYLH	0.1	5.09	-0.5	0.604411	Non-Toxin
AYDPLFYLHHSNTDRLWAI	-0.07	6	0	0.603795	Non-Toxin
TAYDPIFYLH	0.08	5.09	-0.5	0.602852	Non-Toxin
VHTPLKPF	-0.02	9.11	1.5	0.601803	Non-Toxin
DNSADGF	-0.19	3.57	-2	0.601377	Non-Toxin
YSRPIDTF	-0.2	6.19	0	0.590551	Non-Toxin
YASYDPIFYIH	0.08	5.09	-0.5	0.589392	Non-Toxin
NVDRQFAIWQALQKFR	-0.26	10.84	2	0.58748	Non-Toxin
EYTAYDPLF	-0.02	3.67	-2	0.587178	Non-Toxin
GGPTESPWAYDR	-0.23	4.38	-1	0.586936	Non-Toxin
YDDDYHFK	-0.38	4.42	-1.5	0.584445	Non-Toxin
HHNPFHDAEIAFL	-0.02	5.73	-0.5	0.581672	Non-Toxin
HYASYDPLFYLH	0.01	5.99	0	0.581254	Non-Toxin
SYVHTPLKPF	-0.04	8.94	1.5	0.580663	Non-Toxin
LPSDILPHPS	-0.01	5.09	-0.5	0.580608	Non-Toxin
SVLGGELEMPFTF	0.13	3.8	-2	0.579398	Non-Toxin
AVFPHWHRLLTVQ	-0.01	10.11	2	0.579335	Non-Toxin
DYSAFDPFFIIHHS	0.05	5.06	-1	0.578375	Non-Toxin
EMPWTN	-0.15	4	-1	0.574449	Non-Toxin
LLVVQVEDALRRRGSPIGVPY	-0.13	9.1	1	0.573967	Non-Toxin
EYSAYDPIFFLHH	0.02	5.16	-1	0.573514	Non-Toxin
SLEYSAYDPIFFLH	0.07	4.36	-1.5	0.570887	Non-Toxin
WDWTLPIKELPA	0	4.38	-1	0.570111	Non-Toxin
LYTAFDPIFYLH	0.16	5.09	-0.5	0.568811	Non-Toxin
LDYTLYDPIFFLH	0.11	4.2	-1.5	0.567036	Non-Toxin
VGPHGWD	0.01	5.09	-0.5	0.564925	Non-Toxin
VFKPGLPY	0.08	8.94	1	0.563202	Non-Toxin

Peptide	Hydrophobicity (kcal/mol)	pI	Charge at pH7	Peptide Ranker	ToxinPred
NDPEFGDESFFYRQ	-0.29	3.92	-3	0.562896	Non-Toxin
GGPTESPWAY	-0.02	4	-1	0.562011	Non-Toxin
SILGGETEMPFEFDRLF	-0.04	4.01	-3	0.55693	Non-Toxin
DYSAFDPVFIIVH	0.14	4.2	-1.5	0.554811	Non-Toxin
EYTSYDPLFFLH	0.01	4.36	-1.5	0.554662	Non-Toxin
AYDPIFYLHHSNTDRLWAI	-0.06	6	0	0.554577	Non-Toxin
NYQYDDLNFHGL	-0.16	4.2	-1.5	0.553569	Non-Toxin
SLDYSAFDPVFFLHHA	0.07	5.06	-1	0.552753	Non-Toxin
RYDDDYHF	-0.46	4.42	-1.5	0.55207	Non-Toxin
HYASYDPLFYIH	0.03	5.99	0	0.551247	Non-Toxin
SLSYSAFDPIFYIH	0.11	5.09	-0.5	0.550072	Non-Toxin
SSLEYSAYDPIFFLH	0.05	4.36	-1.5	0.548087	Non-Toxin
QLPSDILPHPS	-0.07	5.09	-0.5	0.547339	Non-Toxin
EYTAYDPLFLI	0.1	3.67	-2	0.547043	Non-Toxin
GSHIGIPY	0.13	7.09	0.5	0.542822	Non-Toxin
FFGDSS	0.02	3.8	-1	0.540323	Non-Toxin
LSSLDYTLYDPIFFLH	0.09	4.2	-1.5	0.540274	Non-Toxin
VVPNPFAH	0.09	7.1	0.5	0.540228	Non-Toxin
AIDAFDYDR	-0.23	3.94	-2	0.537916	Non-Toxin
SLSSLDYTLYDPIFFLH	0.07	4.2	-1.5	0.537777	Non-Toxin
EYSAYDPLFFIHH	0.02	5.16	-1	0.537405	Non-Toxin
SLDYAAFDPVFFLHHS	0.07	5.06	-1	0.537128	Non-Toxin
AIPYWDWTSHVSELPK	-0.05	5.33	-0.5	0.536986	Non-Toxin
YDPLFYLHHSNTDRLWAI	-0.09	6	0	0.532688	Non-Toxin
QFAIWQALQK	-0.04	9.11	1	0.529441	Non-Toxin
EYTAYDPLFLLH	0.04	4.36	-1.5	0.527632	Non-Toxin
GGPTESPW	-0.06	4	-1	0.525076	Non-Toxin
SLDYTLYDPIFFLHH	0.06	5.06	-1	0.524982	Non-Toxin
TLDYSAFDPFFIIH	0.12	4.2	-1.5	0.52401	Non-Toxin
EMPWAYER	-0.27	4.54	-1	0.523663	Non-Toxin
APREQLFNDPEFGSESFFYRQ	-0.26	4.41	-2	0.522572	Non-Toxin
DYTAYDPIFFLHH	0.02	5.06	-1	0.521301	Non-Toxin
AVFPHWHRLITVQ	0.01	10.11	2	0.520176	Non-Toxin
HYASYDPL	-0.08	5.09	-0.5	0.519521	Non-Toxin
DYTLYDPIFFLHH	0.04	5.06	-1	0.519472	Non-Toxin
AYDPLFYLHHSNTDRIWAI	-0.06	6	0	0.519358	Non-Toxin
SKEMPWTN	-0.28	6.35	0	0.518019	Non-Toxin
GGELEMPFTFD	0.01	3.58	-3	0.51606	Non-Toxin

Peptide	Hydrophobicity (kcal/mol)	pI	Charge at pH7	Peptide Ranker	ToxinPred
EYSAYDPIFFIHH	0.04	5.16	-1	0.514997	Non-Toxin
PSDILPHPS	-0.07	5.09	-0.5	0.513837	Non-Toxin
YDPIFYLHHSNTDRLWAI	-0.08	6	0	0.512988	Non-Toxin
HHSNVDRQFAIWQALQKFR	-0.27	10.84	3	0.510769	Non-Toxin
NGFQNIASFHGSPAR	-0.13	10.11	1.5	0.510421	Non-Toxin
DLGHPFPDPTVIH	0	5.06	-1	0.509997	Non-Toxin
VFLTFPTAWWLSDAVKNPAFVVK	0.1	8.94	1	0.50795	Non-Toxin
SILGGETEMPFEFDRLFRI	-0.09	4.41	-2	0.506461	Non-Toxin
DYAAYDPVFFLHHT	0.02	5.06	-1	0.506427	Non-Toxin
NLESLSPGEVESLRAAFL	-0.07	4.26	-2	0.506185	Non-Toxin
IGIPYWDWTTAFTALPS	0.14	3.8	-1	0.506183	Non-Toxin
LNYQYDDLNFHGL	-0.11	4.2	-1.5	0.50543	Non-Toxin
SLEYSAYDPLFFIH	0.07	4.36	-1.5	0.505336	Non-Toxin
SLSSLEYSAYDPIFFLH	0.06	4.36	-1.5	0.502888	Non-Toxin
GHPFPDPTVIHE	-0.04	5.16	-1	0.502825	Non-Toxin
EYTSYDPIFFLH	0.02	4.36	-1.5	0.501759	Non-Toxin
KLKEPLKPF	-0.27	9.72	2	0.50168	Non-Toxin
SFTDKIWIVWQELQK	-0.1	6.42	0	0.501019	Non-Toxin
SLEYSAYDPIFFIH	0.08	4.36	-1.5	0.496898	Non-Toxin
SILGGETEMPFEFDRLFR	-0.13	4.41	-2	0.496558	Non-Toxin
LHSWLGGHAK	-0.02	9.11	2	0.494417	Non-Toxin
SVLGGELEMPFTFDRLY	-0.02	4.14	-2	0.493965	Non-Toxin
DYSAFDPV	-0.04	3.57	-2	0.493313	Non-Toxin
GGEKEMPWAYER	-0.3	4.79	-1	0.491569	Non-Toxin
VFKPGLPYT	0.05	8.94	1	0.491135	Non-Toxin
AYDPIFYLHHSNTDRIWAI	-0.05	6	0	0.490342	Non-Toxin
IHSWLGGHAK	0	9.11	2	0.486888	Non-Toxin
SYSAFDPIFYLHHS	0.03	5.99	0	0.485804	Non-Toxin
HSNTDRLFAL	-0.2	7.1	0.5	0.485142	Non-Toxin
HHNPFHD	-0.29	6.28	0.5	0.482909	Non-Toxin
YEYDNLEFNGL	-0.12	3.58	-3	0.482889	Non-Toxin
FFIGGSGTY	0.22	5.88	0	0.481026	Non-Toxin
DYAAFDPV	0.02	3.57	-2	0.480566	Non-Toxin
FHYEYDSLEFNGL	-0.06	4.14	-2.5	0.479845	Non-Toxin
FHYEYDNLEFNGL	-0.09	4.14	-2.5	0.478561	Non-Toxin

Peptide	Hydrophobicity (kcal/mol)	pI	Charge at pH7	Peptide Ranker	ToxinPred
SYNYPIE	-0.12	4	-1	0.478291	Non-Toxin
RYDDDYHFK	-0.53	5.31	-0.5	0.477009	Non-Toxin
AIDELPSTF	0.03	3.67	-2	0.475394	Non-Toxin
SAGEIESLRSAFLA	-0.03	4.54	-1	0.474813	Non-Toxin

Table S4 The 88 small-molecule constituents identified in WJSS.

Number	Name	Average MZ	Adduct type	Total score
1	Piperidine	86.0966	[M+H]+	71
2	Proline	116.07054	[M+H]+	80.7
3	Betaine	118.08598	[M+H]+	89.9
4	4-Hydroxybenzaldehyde	123.04385	[M+H]+	73.4
5	4-Imidazoleacetic acid	127.04999	[M+H]+	92.4
6	Pyroglutamic acid	130.04971	[M+H]+	75
7	Quinoline	130.06483	[M+H]+	82.6
8	D-Pipecolic acid	130.08591	[M+H]+	80.4
9	DL-Pipecolinic acid	130.08598	[M+H]+	70.5
10	Isoleucine	132.10179	[M+H]+	70.2
11	2-Phenylacetamide	136.07524	[M+H]+	80.9
12	3-Formylindole	146.05977	[M+H]+	93
13	Glutamine	147.07607	[M+H]+	84.7
14	Lysine	147.11247	[M+H]+	85.6
15	Glutamic acid	148.05998	[M+H]+	84.4
16	Methionine	150.05792	[M+H]+	73.6
17	Histidine	156.07639	[M+H]+	93.4
18	Phenylalanine	166.08583	[M+H]+	84.8
19	Indole-3-acetamide	175.08636	[M+H]+	86.2
20	Gly-Val	175.10739	[M+H]+	75.7
21	3-Indoleacetic acid	176.07042	[M+H]+	83.7
22	Tyrosine	182.08078	[M+H]+	92.6
23	Phosphocholine	184.07275	[M+H]+	73.8
24	N6-Acetyl-L-lysine	189.12314	[M+H]+	76.8
25	Kynurenic acid	190.04944	[M+H]+	79.6
27	Sedanolide	195.13734	[M+H]+	74.8
28	Ile-Ala	203.13869	[M+H]+	78.2
29	Ala-Ile	203.13872	[M+H]+	71.6
30	Gly-Lys	204.1337	[M+H]+	80.4
31	Val-Pro	215.13873	[M+H]+	89.7
32	Valyl-Valine	217.15417	[M+H]+	79.5
33	Phe-Gly	223.1071	[M+H]+	89.3
34	Carvyl propionate	226.17946	[M+NH4]+	73.2

Number	Name	Average MZ	Adduct type	Total score
35	2,2,6,7-Tetramethylbicyclo[4.3.0]nona-1(9),4-diene-7,8-diol	226.17957	[M+NH4]+	71.8
36	Methyl(2E,6Z)dodecadienoate	228.19527	[M+NH4]+	73.4
37	Ile-Pro	229.15443	[M+H]+	81.6
38	Val-Ile	231.17017	[M+H]+	86.5
39	Thr-Leu	233.14928	[M+H]+	71.1
40	Ile-Thr	233.14935	[M+NH4]+	100
41	Phe-Ala	237.12326	[M+H]+	84.2
42	Lysyl-Proline	244.16544	[M+H]+	72.3
43	Isoleucyl-Leucine	245.18521	[M+H]+	80.4
44	Palmitamide	256.26276	[M+H]+	85.5
45	Isoleucyl-Gamma-glutamate	260.16	[M+H]+	73.5
46	Leu-Lys	260.19684	[M+H]+	89.1
47	Leu-Glu	261.14392	[M+H]+	74.2
48	.alpha.-L-Glu-L-Asp	263.08133	[M+H]+	70.2
49	Phe-Thr	267.13388	[M+H]+	88.9
50	Arg-Pro	272.17133	[M+2H]2+	99.6
51	Muramic acid	274.09125	[M+Na]+	74.8
52	Tyr-Pro	279.13321	[M+H]+	83.9
53	Leu-Phe	279.16971	[M+H]+	87.6
54	Ile-Phe	279.1698	[M+H]+	90.7
55	6,10,14-Trimethyl-5,9,13-pentadecatrien-2-one	280.26239	[M+NH4]+	72.9
56	Oleamide	282.27881	[M+H]+	95.7
57	Stearamide	284.29425	[M+H]+	91.2
58	Leu-Arg	288.2023	[M+H]+	83.4
59	Gln-Phe	294.14456	[M+H]+	79.6
60	Tyr-Asp	297.10788	[M+H]+	82.5
61	Phe-Phe	313.15421	[M+H]+	77.4
62	Docosanamide	340.35696	[M+H]+	93.8
63	Leu-Leu-Leu	358.2699	[M+H]+	71.8
64	Gerberinol	365.10422	[M+H]+	85
65	1,2-Diacylglycerol-LD-PI-pool	118.02875	[M-H]-	70.3

Number	Name	Average MZ	Adduct type	Total score
66	L-Malic acid	133.0132	[M-H]-	79.3
67	4-Hydroxybenzoic acid	137.02339	[M-H]-	70.5
68	Indole-3-carboxyaldehyde	144.04439	[M-H]-	99.8
69	(+/-)-Ethyl 2-hydroxy-2-methylbutyrate	145.08583	[M-H]-	79.3
70	4-Hydroxystyrene	165.05466	[M+FA-H]-	70.1
71	N-Carbamoyl-DL-aspartic acid	175.03539	[M-H]-	73.5
72	Allose	179.05556	[M-H]-	73.1
73	DL-3-(4-Hydroxyphenyl)lactic acid	181.04962	[M-H]-	71.3
74	5-Hydroxyindole-3-acetic acid	190.05037	[M-H]-	69.3
75	Citric acid	191.01886	[M-H]-	71.1
76	(-)-Quinic acid	191.05577	[M-H]-	95.9
77	Methoxyeugenol	193.08614	[M-H]-	76.1
78	N-methyl-4,6,7-trihydroxy-1,2,3,4-tetrahydroisoquinoline	194.08171	[M-H]-	78.2
79	Ala-Leu	201.12416	[M-H]-	85.2
80	Indolelactic acid	204.06607	[M-H]-	80.7
81	Gly-Phe	221.09248	[M-H]-	85.3
82	(10betaH,11xi)-11-Hydroxy-13-nor-6-eremophilen-8-one	221.15405	[M-H]-	76.6
83	Leu-Thr	231.13504	[M-H]-	79.4
84	(1alpha,4beta,5beta)-4-Hydroxy-7(11),10(14)-guaiadien-8-one	233.15445	[M-H]-	81.8
85	Leucyl-Isoleucine	243.17087	[M-H]-	70.1
86	(9S,10E,12S,13S)-9,12,13-Trihydroxy-10-octadecenoic acid	329.23291	[M-H]-	70.7
87	(3beta,22E,24R)-3-Hydroxyergosta-5,8,22-trien-7-one	409.31155	[M-H]-	81.8
88	LysoPE(18:0/0:0)	480.30991	[M-H]-	91.9
